# Mechanochemically-induced glass formation from two-dimensional hybrid organic–inorganic perovskites†

**DOI:** 10.1039/d4sc00905c

**Published:** 2024-04-19

**Authors:** Chumei Ye, Giulio I. Lampronti, Lauren N. McHugh, Celia Castillo-Blas, Ayano Kono, Celia Chen, Georgina P. Robertson, Liam A. V. Nagle-Cocco, Weidong Xu, Samuel D. Stranks, Valentina Martinez, Ivana Brekalo, Bahar Karadeniz, Krunoslav Užarević, Wenlong Xue, Pascal Kolodzeiski, Chinmoy Das, Philip Chater, David A. Keen, Siân E. Dutton, Thomas D. Bennett

**Affiliations:** a Department of Materials Science and Metallurgy, University of Cambridge 27 Charles Babbage Road Cambridge Cambridgeshire CB3 0FS UK tdb35@cam.ac.uk; b Cavendish Laboratory, University of Cambridge J. J. Thomson Avenue Cambridge Cambridgeshire CB3 0HE UK; c Department of Chemistry, University of Liverpool Crown Street Liverpool L69 7ZD UK; d Department of Chemical Engineering and Biotechnology, University of Cambridge Philippa Fawcett Drive Cambridge Cambridgeshire CB3 0AS UK; e Division of Physical Chemistry, Ruđer Bošković Institute Zagreb Croatia; f Anorganische Chemie, Fakultät für Chemie und Chemische Biologie, Technische Universität Dortmund Otto-Hahn-Straße 6 44227 Dortmund Germany; g Department of Chemistry, SRM University-AP Andhra Pradesh-522240 India; h Diamond Light Source Ltd. Diamond House, Harwell Campus Didcot Oxfordshire OX11 0QX UK; i ISIS Facility, Rutherford Appleton Laboratory Harwell Campus Didcot Oxfordshire OX11 0QX UK

## Abstract

Hybrid organic–inorganic perovskites (HOIPs) occupy a prominent position in the field of materials chemistry due to their attractive optoelectronic properties. While extensive work has been done on the crystalline materials over the past decades, the newly reported glasses formed from HOIPs open up a new avenue for perovskite research with their unique structures and functionalities. Melt-quenching is the predominant route to glass formation; however, the absence of a stable liquid state prior to thermal decomposition precludes this method for most HOIPs. In this work, we describe the first mechanochemically-induced crystal-glass transformation of HOIPs as a rapid, green and efficient approach for producing glasses. The amorphous phase was formed from the crystalline phase within 10 minutes of ball-milling, and exhibited glass transition behaviour as evidenced by thermal analysis techniques. Time-resolved *in situ* ball-milling with synchrotron powder diffraction was employed to study the microstructural evolution of amorphisation, which showed that the crystallite size reaches a comminution limit before the amorphisation process is complete, indicating that energy may be further accumulated as crystal defects. Total scattering experiments revealed the limited short-range order of amorphous HOIPs, and their optical properties were studied by ultraviolet-visible (UV-vis) spectroscopy and photoluminescence (PL) spectroscopy.

## Abstract 2

We present the crystal-glass transformation of two-dimensional hybrid organic–inorganic perovskites (HOIPs) *via* ball-milling.

## Graphical abstract

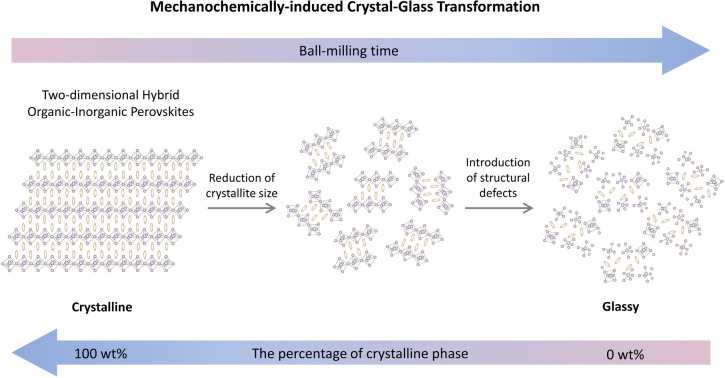

## Introduction

Recently, hybrid glasses derived from hybrid materials, including coordination polymers (CPs), metal–organic frameworks (MOFs) and hybrid organic–inorganic perovskites (HOIPs), have attracted broad interest across materials science.^1–3^ These glassy materials not only inherit chemical diversity and compositional tunability from their crystalline counterparts but also show advantages such as optical transparency and high moldability, as demonstrated in some other glasses.^4^ The intrinsic disorder in hybrid glasses can endow materials with attractive functionalities and show promising applications in areas such as gas adsorption, separation, and ion transport.^5–7^

The most popular approach to forming glasses from hybrid materials is melt-quenching; *i.e.*, heating a crystalline sample above its melting point (*T*_m_), followed by rapid cooling to achieve vitrification. However, this is limited to a few thermally stable hybrid materials that melt prior to decomposition.^1^ Mechanochemistry has recently emerged as a powerful tool for the green synthesis of a variety of solid materials as it reduces or minimises the consumption of organic solvents and thermal energy.^8^ In addition to yielding crystalline materials by grinding or ball-milling solid starting reagents,^9,10^ it also allows for the amorphisation or vitrification of various hybrid materials. Without the need to form stable liquids as in melt-quenching, this direct mechanochemically-induced vitrification method of preparing a glass from a crystal may accordingly be applicable to a wider range of materials, which may form glasses. Thus far, mechanochemically-induced hybrid glasses have been reported in several CPs and MOFs, including phosphate-azole frameworks M^2+^(1,2,4-trizole)_2_(H_2_PO_4_)_2_ (M^2+^ = Cd^2+^, Cr^2+^, Mn^2+^),^11,12^ Ag-tripodal nitrile frameworks [Ag(*m*L1)(CF_3_SO_3_)]·C_6_H_6_ and [Ag(*p*L2)(CF_3_SO_3_)]·C_6_H_6_ (*m*L1 = 1,3,5-tris(3-cyanophenylethynyl)benzene, *p*L2 = 1,3,5-tris(4-cyanophenylethynyl)benzene),^13,14^ Prussian blue analogues,^15^ and zeolitic imidazolate frameworks (ZIFs) ZIF-62 (Zn(Im)_2−*x*_(bIm)_*x*_, Im = imidazolate, bIm = benzimidazolate, *x* = 0.35, 0.5, 1.0).^16^ However, at present, there are no reports involving the vitrification of HOIPs through mechanical milling, though the glasses have been obtained *via* the melt-quenching of three-dimensional [TPrA][M(dca)_3_]^17^ (TPrA^+^ = tetrapropylammonium, M^2+^ = Mn^2+^, Fe^2+^, Co^2+^, dca = dicyanamide) and two-dimensional (2D) (*S*-NEA)_2_PbBr_4_ (*S*-NEA^+^ = (*S*)-(−)-1-(1-naphthyl)ethylammonium)^18^ hybrid perovskites. It should be noted that the HOIPs after amorphisation are not strictly based on the perovskite structure-type.

In this study, we describe the first example of mechanochemically-induced crystal-glass transformation of both a melting and a non-melting HOIP crystal. The effect of milling time on their glassy behaviour was investigated using various thermal techniques. Time-resolved *in situ* synchrotron X-ray diffraction and X-ray total scattering were employed to probe the microstructural evolution of amorphisation, and to compare the difference in local structure between the crystalline and glassy phases. Finally, their optical properties were compared through UV-vis absorption and PL spectroscopy.

## Results and discussion

### Crystal structure and X-ray diffraction

**Fig. 1:**
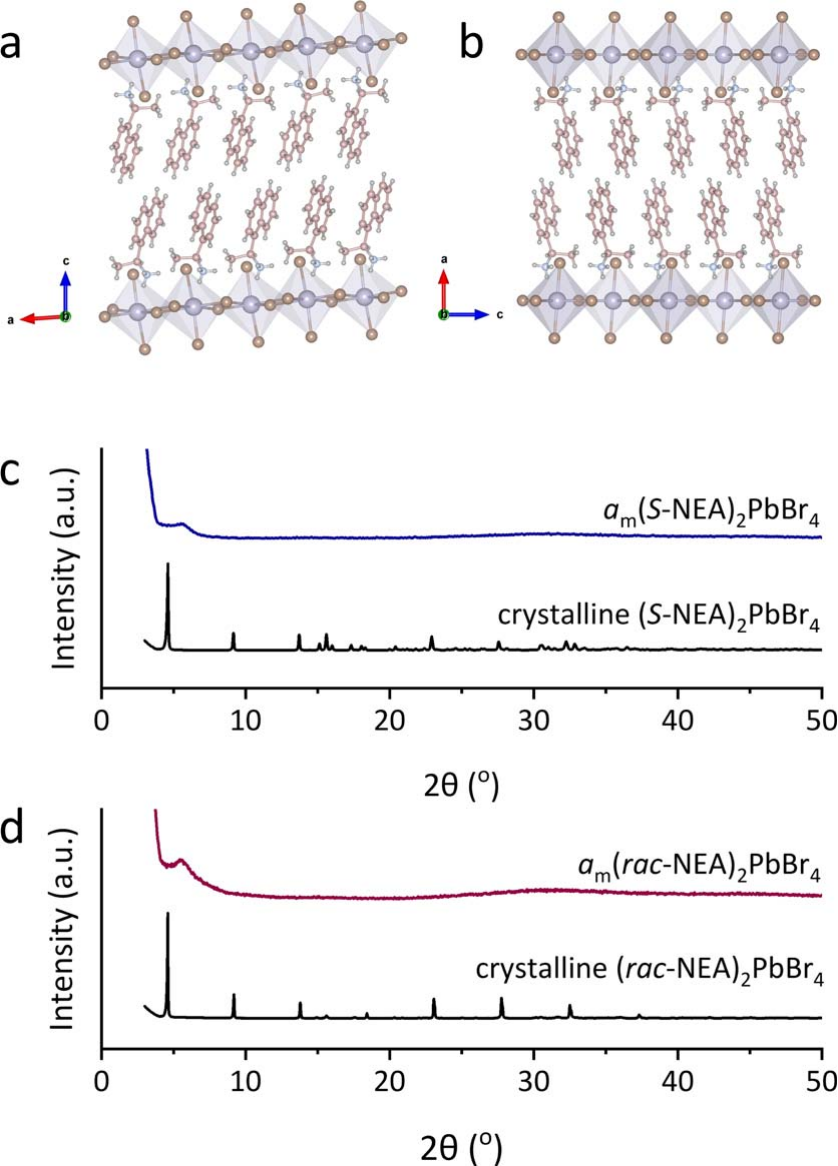
Single crystal structures of (a) (*S*-NEA)_2_PbBr_4_ and (b) (*rac*-NEA)_2_PbBr_4_. Pb, Br, C, N and H atoms are represented by purple, brown, pink, blue, and grey colours, respectively. PXRD patterns of (c) (*S*-NEA)_2_PbBr_4_ and (d) (*rac*-NEA)_2_PbBr_4_ before and after 30 minutes of ball-milling. X-ray wavelength = 1.5418 Å.

Two 2D HOIPs, chiral (*S*-NEA)_2_PbBr_4_ and racemic (1-(1-naphthyl)ethylammonium)_2_PbBr_4_ ((*rac*-NEA)_2_PbBr_4_), were synthesised following the reported methodology.^19^ Both compounds are composed of alternatively stacked organic bilayers and inorganic layers of corner-sharing [PbBr_6_] octahedra (Fig. 1a and b). (*S*-NEA)_2_PbBr_4_ and (*rac*-NEA)_2_PbBr_4_ both crystallise in the monoclinic system, with a space group of *P*2_1_ and *P*2_1_/*c*, respectively. The chiral spacers *S*-NEA^+^ cause symmetry-breaking helical distortions in the inorganic lead bromide framework through asymmetric H-bonding interactions, which are absent in the racemic analogue.^19^ Powder X-ray diffraction (PXRD) was performed on the as-synthesised (*S*-NEA)_2_PbBr_4_ (Fig. 1c) and (*rac*-NEA)_2_PbBr_4_ (Fig. 1d), and their purity was confirmed through Pawley refinements (see Fig. S1, S2, Tables S1 and S2 in ESI†).^20,21^ Fully amorphous *a*_m_(*S*-NEA)_2_PbBr_4_ (*a*_m_ = mechanically amorphised) and *a*_m_(*rac*-NEA)_2_PbBr_4_ were produced after 30 minutes of ball-milling at 30 Hz using one stainless steel ball and exhibit featureless PXRD patterns typical of amorphous materials (Fig. 1c and d).

### Thermal analysis, CHN analysis and ^1^H NMR

**Fig. 2:**
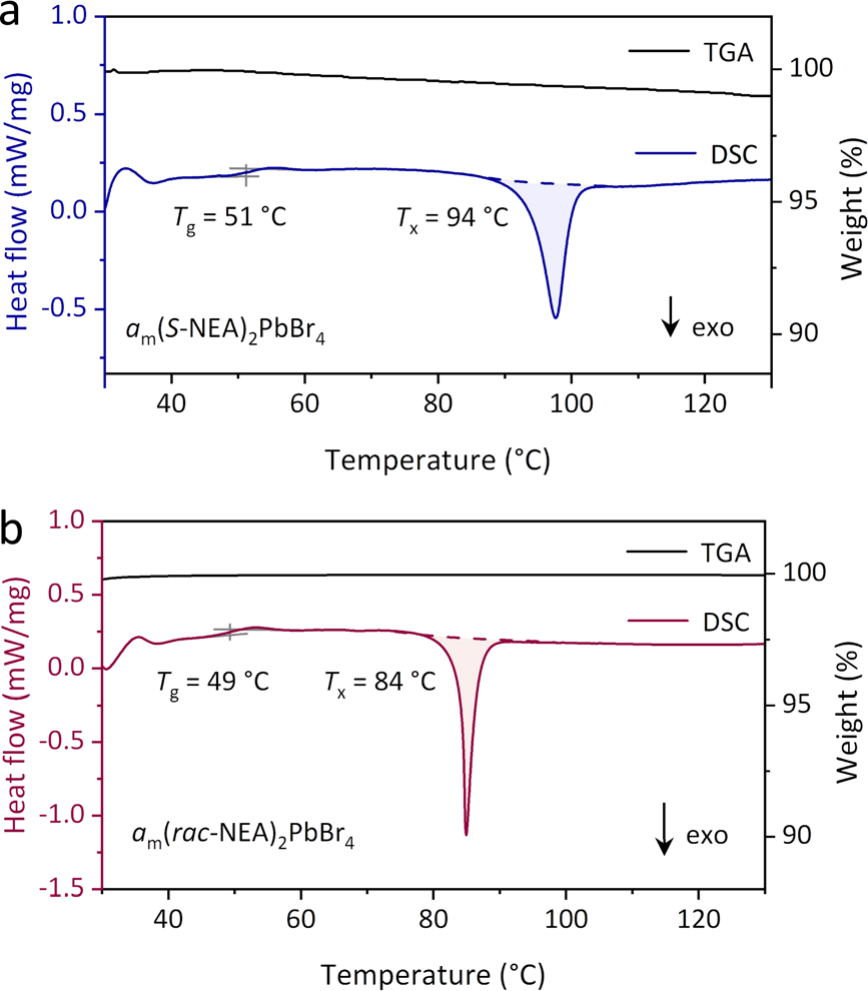
TGA and DSC profiles of (a) *a*_m_(*S*-NEA)_2_PbBr_4_ and (b) *a*_m_(*rac*-NEA)_2_PbBr_4_ in argon. All heating rates are 10 °C min^−1^. *T*_g_ and *T*_*x*_ indicate the glass transition temperature and the recrystallisation temperature, respectively.

Thermogravimetric analysis (TGA) and differential scanning calorimetry (DSC) were further conducted on *a*_m_(*S*-NEA)_2_PbBr_4_ products (Fig. 2a and S3–S5†). No obvious mass loss (<1 wt%) was observed below 200 °C in TGA. The DSC upscan showed typical glass transition behaviour, which is an intrinsic characteristic of a glass and features a reversible transition from a relatively hard and brittle “glassy” phase to a softer and more “liquid-like” phase.^4^ The glass transition temperature (*T*_g_ = 51 °C) of *a*_m_(*S*-NEA)_2_PbBr_4_ was lower than that of a glass sample produced *via* melt-quenching (Fig. S5†).^18^ Similar behaviour has been observed in several CP glasses^14^ and MOF glasses.^16^ Following the glass transition, an exothermic event was observed at 94 °C in the DSC profile (Fig. 2a). PXRD confirmed that it arose from recrystallisation, as the annealed *a*_m_(*S*-NEA)_2_PbBr_4_ is isostructural to the parent crystalline phase (Fig. S6, S7 and Table S3†). It was also observed that *a*_m_(*S*-NEA)_2_PbBr_4_ recrystallised in an ambient environment within 9 hours (Fig. S8–S10 and Tables S4 and S5†). When stored in the freezer at *ca*. 0 °C (Fig. S11a†), or stored under vacuum at room temperature (Fig. S11b†), the recrystallisation process of *a*_m_(*S*-NEA)_2_PbBr_4_ was slowed, though weak Bragg peaks of the crystalline counterpart appeared again after 48 hours. This suggests that storage at low temperature or isolation from moisture (Fig. S12†) is beneficial in hindering the glass-crystal transformation. CHN microanalysis (Table S6†) and ^1^H NMR spectroscopy (Fig. S13†) confirmed that the organic cations (*S*-NEA)^+^ were well preserved in *a*_m_(*S*-NEA)_2_PbBr_4_ upon glass formation. In addition to the chiral HOIP, the glassy phase of the racemic analogue was also observed after ball-milling, which exhibited a *T*_g_ of 49 °C and a recrystallisation temperature (*T*_*x*_) of 84 °C (Fig. 2b and S14†). To our knowledge this is the first report of a glassy phase of (*rac*-NEA)_2_PbBr_4_ as decomposition of the melt prevents glass formation *via* conventional melt-quenching (Fig. S3b†).^18^

### Effect of milling time on glassy behaviour

**Fig. 3:**
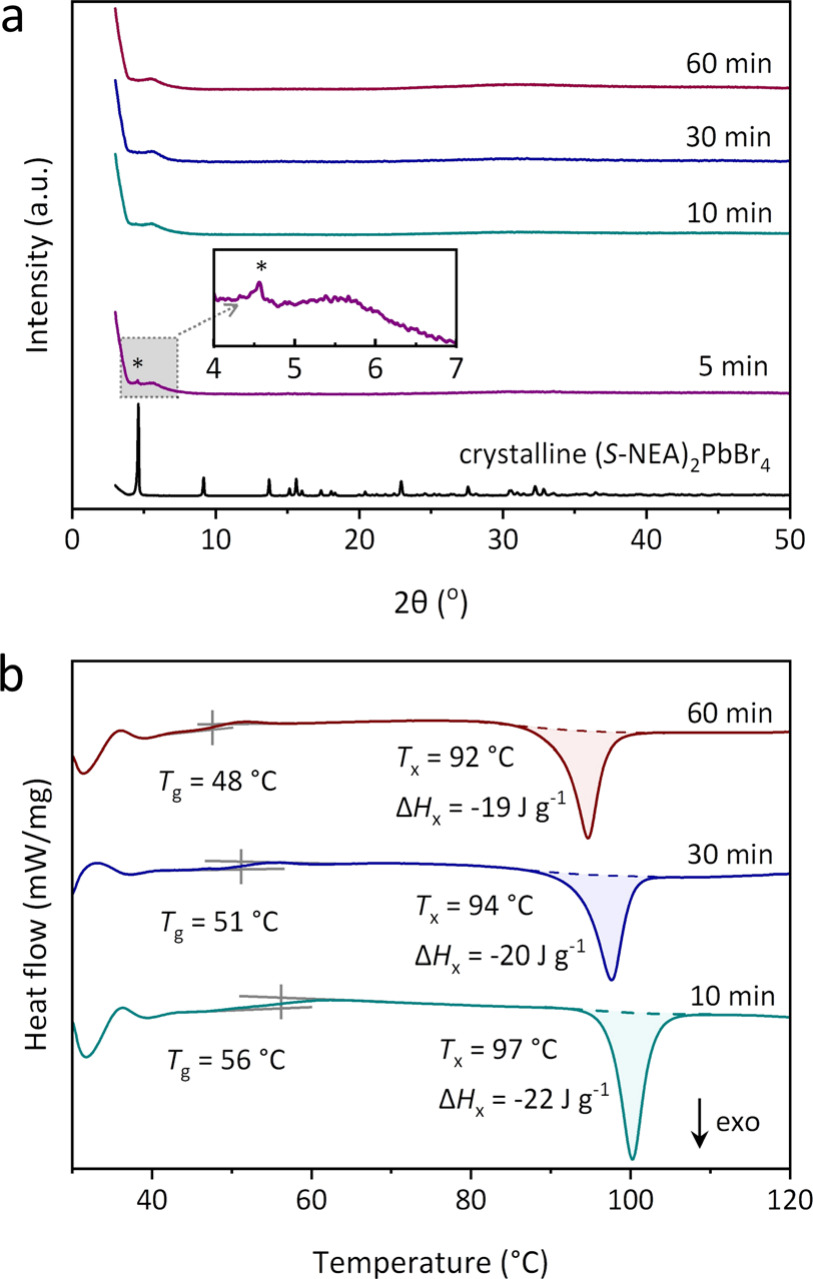
(a) PXRD patterns of crystalline (*S*-NEA)_2_PbBr_4_, *a*_m_(*S*-NEA)_2_PbBr_4_-5, *a*_m_(*S*-NEA)_2_PbBr_4_-10, *a*_m_(*S*-NEA)_2_PbBr_4_-30 and *a*_m_(*S*-NEA)_2_PbBr_4_-60. *The crystalline peak appeared at around 4.6° in *a*_m_(*S*-NEA)_2_PbBr_4_-5. (b) DSC profiles of *a*_m_(*S*-NEA)_2_PbBr_4_-10, *a*_m_(*S*-NEA)_2_PbBr_4_-30 and *a*_m_(*S*-NEA)_2_PbBr_4_-60 in argon. The ramping rate is 10 °C min^−1^. Δ*H*_*x*_ is the difference in enthalpy of recrystallisation.

To examine the effect of ball-milling time on the thermal behaviour of *a*_m_(*S*-NEA)_2_PbBr_4_, ex situ ball-milling treatments were performed on crystalline (*S*-NEA)_2_PbBr_4_ powders for 5, 10, 30 and 60 minutes. The products are thus denoted as *a*_m_(*S*-NEA)_2_PbBr_4_-*x*, where *x* is the milling time in minutes. A weak crystalline peak at 4.6° was retained in the PXRD of *a*_m_(*S*-NEA)_2_PbBr_4_-5 (Fig. 3a), suggesting that the amorphisation was not completed within 5 minutes. However, after 10 minutes of ball-milling, only diffuse scattering was observed, supporting its full transformation to the amorphous phase. Additionally, the product remained amorphous after 60 minutes of ball-milling with no further phase changes. DSC measurements showed that the *T*_g_s of *a*_m_(*S*-NEA)_2_PbBr_4_-10, *a*_m_(*S*-NEA)_2_PbBr_4_-30 and *a*_m_(*S*-NEA)_2_PbBr_4_-60 were 56 °C, 51 °C and 48 °C, respectively (Fig. 3b). This decreasing *T*_g_ likely arises from a weaker network connectivity upon longer milling time.^22^ The recrystallisation temperature and the difference in enthalpy of recrystallisation (Δ*H*_*x*_) are 97 °C and −22 ± 1 J g^−1^ (*a*_m_(*S*-NEA)_2_PbBr_4_-10), 94 °C and −20 ± 1 J g^−1^ (*a*_m_(*S*-NEA)_2_PbBr_4_-30), 92 °C and −19 ± 1 J g^−1^ (*a*_m_(*S*-NEA)_2_PbBr_4_-60), respectively (Fig. 3b and S15†). FTIR spectra of crystalline (*S*-NEA)_2_PbBr_4_ and *a*_m_(*S*-NEA)_2_PbBr_4_-*x* with different milling times were collected from 4000–550 cm^−1^ to confirm the presence of the organic (*S*-NEA)^+^ cation in the structure. They showed identical absorbance, including N–H and C–H stretching at around 3000 cm^−1^, aromatic C=C stretching at 1580 cm^−1^ and C–H bending at 770 cm^−1^, indicating that no new chemical bonds were formed during the milling process, and that the structure of the organic cations remained intact (Fig. S16†).

### Microstructural evolution upon ball-milling

**Fig. 4:**
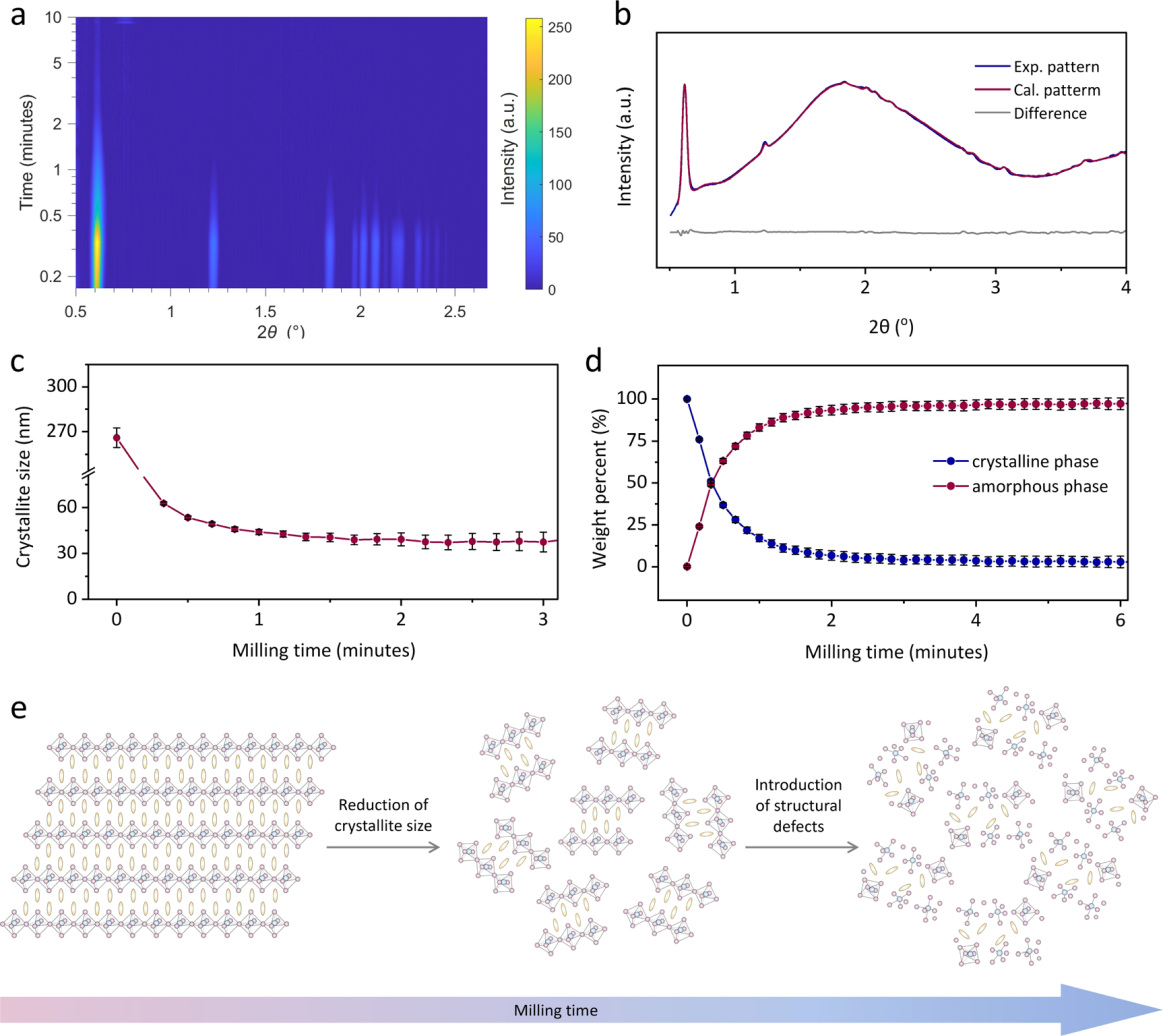
Time-resolved *in situ* (TRIS) ball-milling powder X-ray diffraction. (a) Time-resolved diffractograms for the mechanically-induced amorphisation of (*S*-NEA)_2_PbBr_4_. (b) A Rietveld fit example using the diffractogram collected at PETRA-III in the 10^th^ second, showing calculated (red curve), experimental (blue curve), and difference (grey curve) patterns. (c) Scherrer crystallite size and (d) weight percent quantification for the crystalline (blue) and the amorphous (red) fractions, with their relative estimated standard deviations as error bars as obtained from Rietveld analysis of the TRIS PXRD. Solid lines in (c) and (d) connect data points as a guide to the eye. (e) Schematic illustration of the microstructural evolution on 2D HOIPs upon ball-milling.

To further illustrate the mechanochemically-induced amorphisation in 2D HOIPs, time-resolved *in situ* (TRIS) ball-milling synchrotron PXRD data was collected at the PETRA III beamline P02.1 in DESY (Fig. 4).^23^ It revealed that crystalline (*S*-NEA)_2_PbBr_4_ took *ca.* 5 minutes to fully amorphise (Fig. 4a and S17†), which was quicker than amorphisation under laboratory milling (Fig. 3a). This is attributed to the slightly different milling conditions. For example, the *in situ* measurements were performed in a PMMA milling jar (Fig. S18†) to allow the penetration of X-ray beams, while conventional laboratory ball-milling experiments used stainless steel milling jars. The subsequent Rietveld refinements^24^ were carried out on the diffraction data using TOPAS Academic V7 (ref. 21) to demonstrate the microstructural evolution (Fig. S19–S22†).^25,26^ Details of the refinement strategy are reported in the ESI.† The sample used in the *in situ* experiment was found to contain a minor impurity (<2% in weight) of lead oxide (Pb_3_O_4_), probably an impurity from the synthesis. This did not change in abundance during the experiment nor did it affect the amorphisation process. Fig. 4b shows an example of a Rietveld refinement, indicating the quality of the fit. During measurements, the Scherrer crystal size decreased rapidly from *ca.* 300 nm to reach what looks to be a comminution average crystallite size limit of *ca.* 40 nm within 20 seconds, when the amorphous fraction is less than 50 wt% (Fig. 4c and d).^26,27^ Milling causes a decrease in the crystal quality in the powder, initially through crystal comminution with an increase in crystal surface energy. At some comminution limit,‡‡Communition limit: particles of brittle materials are expected to become smaller with longer milling time until a brittle–ductile transition occurs, as there is no longer sufficient stored energy in the particles to initiate and further propagate cracks throughout the particles.^36^ further breakage is not possible, we accordingly hypothesise that the excess energy is stored in the nanocrystallites as structural defects (Fig. 4e). The accumulation of defects in the crystallites generally results in microstrain contribution to peak broadening, which is expected to be more significant at higher 2*θ* angle compared to the contribution of crystal size. Unfortunately, the weak signal of the reflections in the higher angle range did not allow for reliable estimates of the microstrain contribution to peak broadening beyond the first few scans. Following complete amorphisation, further heating towards *ca.* 80 °C resulted in rapid recrystallisation of *a*_m_(*S*-NEA)_2_PbBr_4_ within 5 minutes (at *ca.* 50 °C, see Fig. S23†), highlighting the strong tendency for *a*_m_(*S*-NEA)_2_PbBr_4_ to recrystallise.

### Local structure analysis from pair distribution functions

**Fig. 5:**
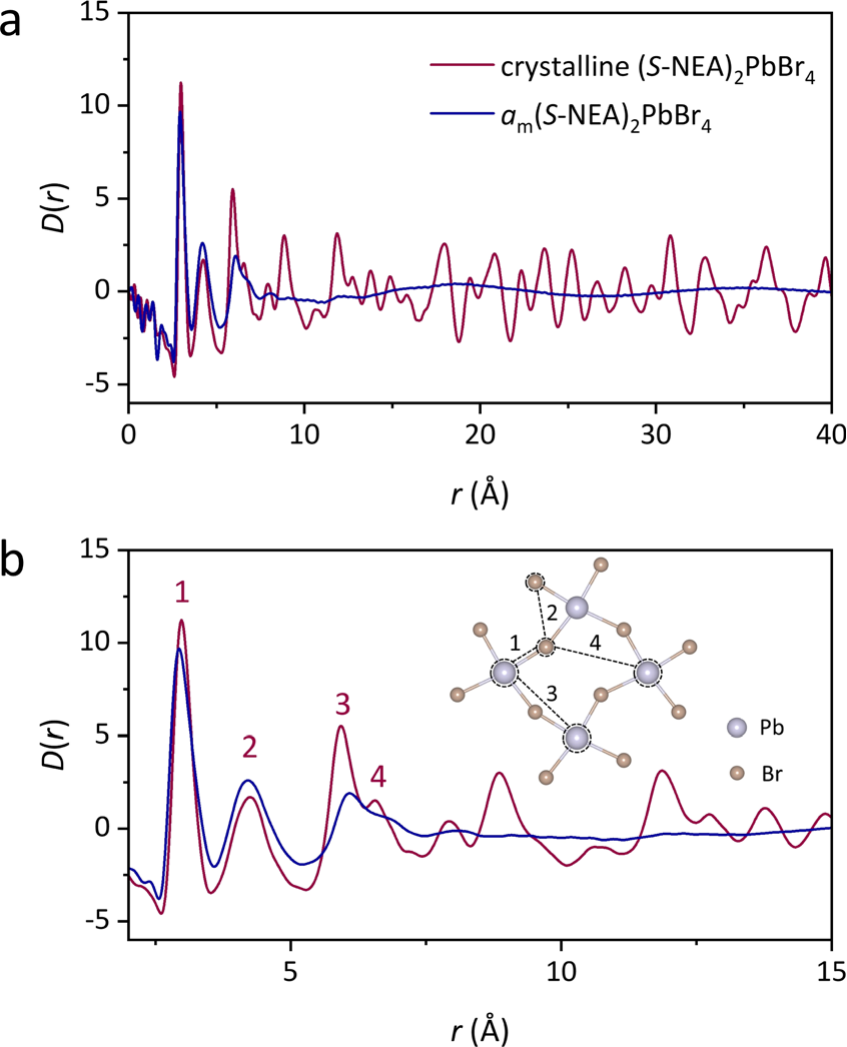
PDFs of crystalline (*S*-NEA)_2_PbBr_4_ (red) and *a*_m_(*S*-NEA)_2_PbBr_4_ (blue). (a) *D*(*r*) between 0 and 40 Å, highlighting the absence of long-range order in *a*_m_(*S*-NEA)_2_PbBr_4_. (b) *D*(*r*) between 0 and 15 Å, showing the similarities in local structure between crystalline (*S*-NEA)_2_PbBr_4_ and *a*_m_(*S*-NEA)_2_PbBr_4_. The correlations below 7 Å are labelled from 1–4, which are shown in the inset.

The atomic structures of crystalline (*S*-NEA)_2_PbBr_4_ and the corresponding mechanochemically-induced glass were probed *via* synchrotron X-ray total scattering conducted at beamline I15-1 at the Diamond Light Source. The absence of crystallinity in the amorphous HOIP is evidenced by the lack of sharp features in the structure factor (Fig. S24†). Pair distribution functions (PDFs) of crystalline (*S*-NEA)_2_PbBr_4_ and *a*_m_(*S*-NEA)_2_PbBr_4_ were obtained from the Fourier transformation of the corrected total scattering data (Fig. 5). As expected, the crystalline HOIP contains long-range oscillations in the *D*(*r*) on length scales above 10 Å, whereas the glassy sample PDF is relatively featureless in this region (Fig. 5a). The lack of distinct correlations over longer distances in *a*_m_(*S*-NEA)_2_PbBr_4_ confirms its transformation to the glass phase.

At low-*r*, the PDF profiles for crystal and glass are similar below 5 Å (Fig. 5b). Peak assignment was carried out using PDFgui,^28^ where partial PDFs for atom pairs were calculated based on the crystal structure (Fig. S25†). Note that correlations involving strongly-scattering Pb (and to a lesser extent Br) atoms tend to dominate the PDF signal. The most intense feature at 3 Å (labelled 1 in Fig. 5b) is assigned to the nearest Pb–Br distance, while the second peak at 4.2 Å (labelled 2 in Fig. 5b) corresponds to Br–Br interactions within the same [PbBr_6_]^2−^ octahedron. Correlations 3 to 4 are dominated by Pb–Pb and Pb–Br interactions between two neighbouring octahedra, respectively. These features reveal that the limited short-range order is preserved after the crystal-glass phase transition of HOIPs.

### Optical properties

**Fig. 6:**
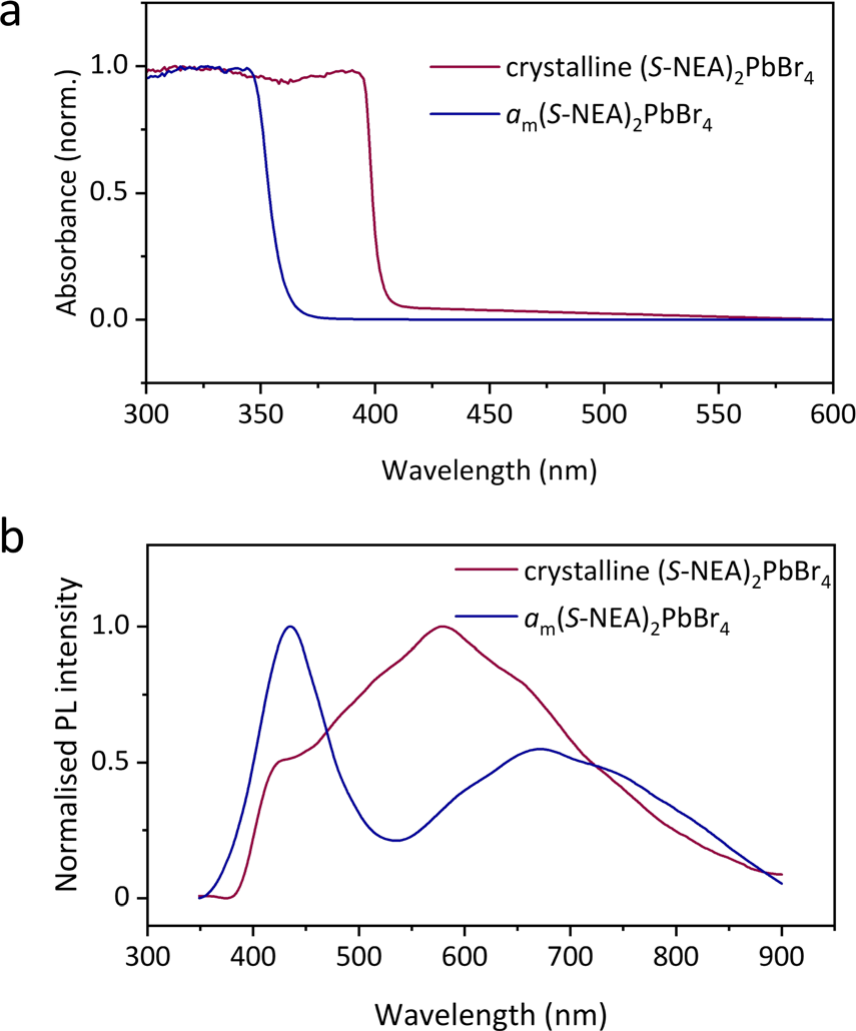
Optical properties of crystalline (*S*-NEA)_2_PbBr_4_ (red) and *a*_m_(*S*-NEA)_2_PbBr_4_ (blue). (a) UV-vis absorption spectra recorded at ambient temperature. (b) Steady-state photoluminescence spectra obtained at ambient temperature using a 300 nm light source.

The optical properties of both crystalline HOIPs and *a*_m_HOIPs were studied by UV-vis absorption and PL spectroscopy at ambient temperature (Fig. 6). The absorption onset is located at approximately 400 nm for crystalline (*S*-NEA)_2_PbBr_4_ powders at ambient temperature (Fig. 6a), which is in accordance with the reported result for the crystalline film on soda lime glass.^18^ It shifts to approximately 360 nm for *a*_m_(*S*-NEA)_2_PbBr_4_, suggesting that the mechanochemically-induced crystal-glass transformation results in a significant change of the bandgap structure. The PL spectra of crystalline (*S*-NEA)_2_PbBr_4_ exhibits a broad emission at around 600 nm (Fig. 6b), consistent with the reported literature.^19^ It may originate from self-trapped excitonic emission^19^ or halide vacancies,^29^ which has been commonly observed in two-dimensional hybrid perovskites.^30–32^ In comparison to the crystalline phase, the PL emission of *a*_m_(*S*-NEA)_2_PbBr_4_ shows a significant shift in wavelength, agreeing well with the absorption spectra (Fig. 6a), while the intensity decreases drastically upon mechanochemically-induced crystal-glass transformation (Fig. S26†).

## Conclusions

In conclusion, we demonstrate the formation of amorphous *a*_m_(*S*-NEA)_2_PbBr_4_ and *a*_m_(*rac*-NEA)_2_PbBr_4_*via* ball-milling. These *a*_m_HOIPs show clear glass transitions and recrystallisation upon heating despite not being melt-quenched. The relatively low *T*_g_-values of *a*_m_HOIPs point to greater glass processability, which opens exciting new opportunities for their industrial implementation. Time-resolved *in situ* synchrotron powder X-ray diffraction monitored the microstructural evolution of amorphisation, indicating that during the milling process, the crystallites reach a crystallite size comminution limit well before the amorphisation process reaches completion. Further energy may be accumulated as structural defects. The *a*_m_(*S*-NEA)_2_PbBr_4_ retains limited short-range order as determined by pair distribution function measurements. UV-vis absorption study and photoluminescence spectroscopy show that the optical properties of the glasses formed from HOIPs are significantly different from those of their crystalline counterparts. This mechanochemically-induced amorphisation approach to achieving the crystal-glass transformation of HOIPs circumvents the requirement to melt-quench a crystalline material to form a glass. It provides a promising case study, illustrating that ball-milling may be applied to a wide variety of glass-forming systems in addition to conventional coordination polymers and metal–organic frameworks.

## Experimental

### Materials

Lead(ii) bromide (99.999%) was purchased from Aldrich. (*S*)-(−)-1-(1-naphthyl)ethylamine (99%) and 1-(1-naphthyl)ethylamine (99%) were purchased from Thermo Fisher and Fluorochem, respectively. Hydrobromic acid (47 wt% HBr in H_2_O) was purchased from VMR Chemicals. Diethyl ether (99.8+%) was purchased from Sigma-Aldrich. Methanol was purchased from Fisher Scientific. All reagents were used without further purification.

### Synthesis of chiral (*S*-NEA)_2_PbBr_4_

PbBr_2_ (0.24 mmol, 90 mg) and (*S*)-(−)-1-(1-naphthyl)ethylamine (0.48 mmol, 78 μL) were dissolved in a mixed solution of hydrobromic acid (1.0 mL) and deionized water (2.4 mL) at 95 °C in a sealed vial. The resultant solution was gradually cooled down to room temperature over a period of two days to obtain crystals with flake-like morphologies, which were then filtered, washed with diethyl ether and dried in vacuum at 90 °C for 12 hours. This synthetic method was designed based on the reported synthesis of (*S*-NEA)_2_PbBr_4_.^18^

### Synthesis of racemic (*rac*-NEA)_2_PbBr_4_

Crystals of (*rac*-NEA)_2_PbBr_4_ were grown in a similar way from a solution of PbBr_2_ (90 mg, 0.24 mmol) and 1-(1-naphthyl)ethylamine (78 μL, 0.48 mmol) in hydrobromic acid (1.0 mL) and methanol (2.4 mL).

### Powder X-ray diffraction (PXRD)

Finely ground samples were compacted into 5 mm flat plate discs and data were collected on the Bruker D8 ADVANCE, using CuKα radiation (*λ* = 1.5418 Å) as the X-ray source. Measurements were carried out at room temperature over the 2*θ* range of 3° to 70° for all materials, with a step size of 0.02° and measurement time of 0.750 seconds per step. Pawley refinements^20^ were performed using TOPAS-Academic Version 7.^21^ The lattice parameters were refined over the 2*θ* range of 3–70° against the values obtained from the published Crystallographic Information Files CCDC 2015618 for (*S*-NEA)_2_PbBr_4_ and CCDC 2015614 for (*rac*-NEA)_2_PbBr_4_.^19^

### Lab ball-milling experiments

Approximately 200 mg of finely ground samples were placed in a 10 mL stainless steel ball-mill jar with one 10 mm stainless steel ball of around 4 g. The powders were ball-milled at 30 Hz for different durations including 5, 10, 30 and 60 minutes at room temperature. After the ball-milling process finished, samples were immediately taken out for further measurements.

### Time-resolved *in situ* (TRIS) ball-milling powder X-ray diffraction

*In situ* synchrotron PXRD experiments were performed at PETRA III beamline P02.1 (*λ* = 0.207351 Å), DESY Germany. Data was collected on a Varex XRD4343CT detector, and milling was conducted using a modified IST-636 ball mill, controlled remotely from outside the experimental hutch. Beam alignment and calibration were performed using a Si standard in a poly(methyl methacrylate) (PMMA) milling jar. Data was processed by removing the amorphous background contribution from PMMA. To investigate the amorphisation process, around 100 mg finely ground (*S*-NEA)_2_PbBr_4_ powders were placed into a 10 mL PMMA jar with one 10 mm stainless-steel ball of around 4 g and then milled at 30 Hz for around 40 minutes at room temperature. Following this, the milling ball was removed, and a heating jacket was subsequently attached to the jar to observe recrystallisation.

### TRIS ball-milling PXRD data analysis

To obtain the classic one-dimensional PXRD pattern, the collected two-dimensional diffraction images were integrated with the DAWN Science package. Time-resolved diffractograms were generated by MATLAB R2023a using the MATLAB scripts developed by Dr Stipe Lukin. The structure was first optimized *via* Rietveld refinement using laboratory powder diffraction data with TOPAS Academic V7 (see ESI† for details). Determination of the instrumental resolution function and sequential Rietveld refinements were then performed according to the strategies previously described.^25,26^ Details of the sequential refinements are described in the ESI.† Raw data and sequential input files for TOPAS are supplied as ESI.†

### Thermogravimetric analysis (TGA)

TGA measurements were performed on a METTLER TOLEDO TGA2. Approximately 2–5 mg of evacuated samples were placed on an alumina crucible. Data were collected under argon in the range of 30–800 °C with a ramp rate of 10 °C min^−1^. Data analysis was performed using the TA Instruments Universal Analysis software package.

### Differential scanning calorimetry (DSC)

DSC experiments were performed on a NETZSCH DSC 214 Polyma and data were processed by the Proteus Analysis software. Approximately 2–5 mg of samples were placed into an aluminium crucible with a pierced lid, compressed by a hand press kit and situated at the sample position in DSC. An empty aluminium crucible was used as a reference. Samples were heated to 190 °C at 10 °C min^−1^ unless otherwise stated. *T*_m_ was taken as the onset of the melting endotherm, while *T*_g_ was taken as the mid-point of the change in gradient of the heat flow.

### Simultaneous TGA-DSC (SDT)

SDT measurements were performed on a TA Instruments SDT-Q65. Approximately 2–5 mg of evacuated samples were placed on an alumina crucible. Data were collected under argon in the range of 30–800 °C with a ramp rate of 10 °C min^−1^.

### CHN microanalysis

CHN combustion analysis experiments were performed using a CE440 Elemental Analyser, EAI Exeter Analytical Inc. It was operated with the tolerances of ±0.2% for the C and ±0.1% for the H and N. Approximately 2–5 mg of sample was used for each run, and two measurements were collected per sample.

### Fourier-transform infrared spectroscopy (FT-IR)

FT-IR measurements on powder samples were performed on a Bruker Tensor 27 FTIR spectrometer. Data were collected in transmission mode between 600 cm^−1^ and 4000 cm^−1^ at room temperature.

### ^1^H nuclear magnetic resonance spectroscopy (^1^H NMR)

^1^H NMR experiments were performed in a Bruker Advance III HD 500 MHz spectrometer at room temperature. Around 1–5 mg samples of *a*_m_(*S*-NEA)_2_PbBr_4_ were dissolved in DMSO-d_6_ solution and sonicated for 5 minutes to allow for complete dissolution. Spectra were calibrated using TMS as a standard. Data were processed using MestReNova V14.0.0.

### X-ray total scattering and pair distribution function (PDF)

X-ray total scattering data were collected at beamline I15-1, Diamond Light Source, UK (EE20038) on crystalline (*S*-NEA)_2_PbBr_4_ and *a*_m_(*S*-NEA)_2_PbBr_4_ samples. Finely ground samples were loaded into borosilicate glass capillaries (0.78 mm inner diameter) to 3.68 cm for (*S*-NEA)_2_PbBr_4_ and 3.70 cm for *a*_m_(*S*-NEA)_2_PbBr_4_. The filled capillaries were then sealed with clay before being mounted onto the beamline. Total scattering data were collected at ambient temperature for the background (*i.e.*, empty instrument), empty borosilicate capillary and for both samples in a *Q* range of 0.4–26 Å^−1^ (*λ* = 0.161669 Å). The total scattering data were processed to account for absorption corrections and various scattering corrections (background scattering, multiple scattering, container scattering and Compton scattering) in a *Q* range of 0.4–20 Å^−1^. Subsequent Fourier transformation of the processed total scattering data resulted in a real space pair distribution function *G*(*r*) for each material. In this work, we use the *D*(*r*) form of the pair distribution function to accentuate high *r* correlations. All total scattering data were processed using GudrunX software following well documented procedures.^33–35^

### Ultraviolet-visible (UV-vis) spectroscopy

UV-vis experiments were performed on crystalline (*S*-NEA)_2_PbBr_4_ and *a*_m_(*S*-NEA)_2_PbBr_4_ at ambient temperature using an Agilent UV-vis spectrophotometer, in the range 200–800 nm. Finely ground samples were sandwiched between two glass slides and then sealed using glue for measurements. Two clean glass slides without samples were also sealed together, and its spectra was taken as background.

### Steady-state photoluminescence (PL) spectroscopy

PL spectra of crystalline (*S*-NEA)_2_PbBr_4_ and *a*_m_(*S*-NEA)_2_PbBr_4_ were recorded on the FLS1000 fluorescence spectrometer (Edinburgh Instruments) at room temperature. Excitation was achieved using 300 nm monochromatic light generated from a xenon arc lamp. The resulting PL spectra were acquired using a double-grating Czerny–Turner monochromator and a photomultiplier tube detector. Finely ground samples were sandwiched between two quartz slides and then sealed using clamps for measurements.

## Data availability

The datasets supporting this article have been uploaded as part of the ESI.†

## Author contributions

C. Y. conceptualised and designed the project. C. Y. synthesised all the materials. V. M., B. K., C. Y., I. B. and K. U. collected the *in situ* ball-milling powder X-ray diffraction data and G. I. L. analysed the data. P. K., W. X. (Technische Universität Dortmund), C. D., C. Y. and T. D. B. collected the X-ray total scattering data, while C. Y. analysed the data with inputs from C. C. B., P. C. and D. A. K., C. Y. and W. X. (University of Cambridge) collected the photoluminescence spectra. C. Y. performed and analysed all other experiments. L. N. M., A. K., C. C., G. P. R. and L. N. C. contributed with useful discussions. S. E. D. and T. D. B. supervised the project and acquired funding. C. Y. wrote the manuscript with input from all authors.

## Conflicts of interest

There are no conflicts to declare.

## Supplementary Material

SC-015-D4SC00905C-s001

SC-015-D4SC00905C-s002
