# Supplementary material for: Mechanochemically-induced glass formation from two-dimensional hybrid organic–inorganic perovskites
Source: Chem Sci. 2024 Apr 19;15(19):7198–205. doi: 10.1039/d4sc00905c (PMC11095504; doi:10.1039/d4sc00905c)
Supplement: SC-015-D4SC00905C-s001 [file SC-015-D4SC00905C-s001.pdf]

Electronic Supplementary Material (ESI) for Chemical Science.
This journal is © The Royal Society of Chemistry 2024

Supplementary Information

Mechanochemically-induced glass formation from two-dimensional hybrid organic-inorganic perovskites

Chumei Ye[a, b], Giulio I. Lampronti[a], Lauren N. McHugh[c], Celia Castillo-Blas[a], Ayano Kono[a], Celia Chen[a, b], Georgina P. Robertson[a], Liam A. V. Nagle-Cocco[b], Weidong Xu[d], Samuel D. Stranks[b, d], Valentina Martinez[e], Ivana Brekalo[e], Bahar Karadeniz[e], Krunoslav Užarević[e], Wenlong Xue[f], Pascal Kolodzeiski[f], Chinmoy Das[f, g], Philip Chater[h], David A. Keen[i], Siân E. Dutton[b], Thomas D. Bennett[a]*

[a] Department of Materials Science and Metallurgy, University of Cambridge, 27 Charles Babbage Road, Cambridge, Cambridgeshire, CB3 0FS, UK.

*E-mail: tdb35@cam.ac.uk

[b] Cavendish Laboratory, University of Cambridge, J. J. Thomson Avenue, Cambridge, Cambridgeshire, CB3 0HE, UK.

[c] Department of Chemistry, University of Liverpool, Crown Street, Liverpool, L69 7ZD, UK.

[d] Department of Chemical Engineering and Biotechnology, University of Cambridge, Philippa Fawcett Drive, Cambridge, Cambridgeshire, CB3 0AS, UK.

[e] Division of Physical Chemistry, Ruđer Bošković Institute, Zagreb, Croatia.

[f] Anorganische Chemie, Fakultätfür Chemie & Chemische Biologie, Technische Universität Dortmund, Otto-Hahn-Straße 6, 44227 Dortmund, Germany.

[g] Department of Chemistry, School of Engineering and Sciences, SRM University-AP, Andhra Pradesh-522240, India.

[h] Diamond Light Source Ltd., Diamond House, Harwell Campus, Didcot, Oxfordshire, OX11 0QX, UK.

[i] ISIS Facility, Rutherford Appleton Laboratory, Harwell Campus, Didcot, Oxfordshire, OX11 0QX, UK.

Contents List

Figures

Fig. S1. Pawley refinement of the PXRD for crystalline (*S*-NEA)$_2$PbBr$_4$. 4

Fig. S2. Pawley refinement of the PXRD for crystalline (*rac*-NEA)$_2$PbBr$_4$. 5

Fig. S3. Simultaneous DSC-TGA of (*S*-NEA)$_2$PbBr$_4$ and (*rac*-NEA)$_2$PbBr$_4$ in argon. 6

Fig. S4. TGA profiles of (*S*-NEA)$_2$PbBr$_4$ and (*rac*-NEA)$_2$PbBr$_4$ in argon. 7

Fig. S5. Full DSC scan of a_m(*S*-NEA)$_2$PbBr$_4$. 8

Fig. S6. PXRD pattern of crystalline (*S*-NEA)$_2$PbBr$_4$ and annealed a_m(*S*-NEA)$_2$PbBr$_4$. 9

Fig. S7. Pawley refinement of the PXRD for annealed (*S*-NEA)$_2$PbBr$_4$. 10

Fig. S8. PXRD pattern of a_m(*S*-NEA)$_2$PbBr$_4$ stored in an ambient environment over a period of time. 11

Fig. S9. Pawley refinement of the PXRD for recrystallised a_m(*S*-NEA)$_2$PbBr$_4$ after 9 hours stored in an ambient environment. 12

Fig. S10. Pawley refinement of the PXRD for recrystallised a_m(*S*-NEA)$_2$PbBr$_4$ after 27 hours stored in an ambient environment. 13

Fig. S11. PXRD pattern of a_m(*S*-NEA)$_2$PbBr$_4$ when stored in the freezer or under vacuum. 14

Fig. S12. TGA and DSC profiles of the recrystallised a_m(*S*-NEA)$_2$PbBr$_4$ after two days stored in an ambient environment. 15

Fig. S13. ^{1}H nuclear magnetic resonance spectrum of a_m(*S*-NEA)$_2$PbBr$_4$. 17

Fig. S14. Full DSC scan of a_m(*rac*-NEA)$_2$PbBr$_4$. 18

Fig. S15. Full DSC scan of a_m(*S*-NEA)$_2$PbBr$_4$ with different milling time. 19

Fig. S16. FT-IR of (*S*-NEA)$_2$PbBr$_4$ and a_m(*S*-NEA)$_2$PbBr$_4$ with different milling time. 20

Fig. S17. Full time-resolved in-situ ball-milling PXRD of (*S*-NEA)$_2$PbBr$_4$. 25

Fig. S18. PXRD pattern of the PMMA milling jar 26

Fig. S19. An example Rietveld fit to the PXRD for (*S*-NEA)$_2$PbBr$_4$ under ball-milling at time 0. 27

Fig. S20. An example Rietveld fit to the PXRD for (*S*-NEA)$_2$PbBr$_4$ under ball-milling after 8 minutes. 28

Fig. S21. The initial structural Rietveld refinement of (S-NEA)$_2$PbBr$_4$. 29

Fig. S22. R_{wp}-values and chi-square obtained from Rietveld analysis of the TRIS PXRD for (*S*-NEA)$_2$PbBr$_4$. 30

Fig. S23. Full time-resolved PXRD of a_m(*S*-NEA)$_2$PbBr$_4$ upon heating. 31

Fig. S24. Structure factor *S*(*Q*) of (*S*-NEA)$_2$PbBr$_4$ and a_m(*S*-NEA)$_2$PbBr$_4$. 32

Fig. S25. Selected partial PDFs for crystalline (*S*-NEA)$_2$PbBr$_4$. 33

Fig. S26. Photoluminescence spectra of (*S*-NEA)$_2$PbBr$_4$ and a_m(*S*-NEA)$_2$PbBr$_4$. 34

Tables

Table S1 Crystallographic data from Pawley refinement of the PXRD for (*S*-NEA)$_2$PbBr$_4$. 4

Table S2 Crystallographic data from Pawley refinement of the PXRD for (*rac*-NEA)$_2$PbBr$_4$. 5

Table S3 Crystallographic data from Pawley refinement of the PXRD for annealed (*S*-NEA)$_2$PbBr$_4$. 10

Table S4 Crystallographic data from Pawley refinement of the PXRD for recrystallised (*S*-NEA)$_2$PbBr$_4$ after 9 hours stored in an ambient environment. 12

Table S5 Crystallographic data from Pawley refinement of the PXRD for recrystallised (*S*-NEA)$_2$PbBr$_4$ after 27 hours stored in an ambient environment. 13

Table S6 CHN microanalysis of (*S*-NEA)$_2$PbBr$_4$ and a_m(*S*-NEA)$_2$PbBr$_4$. 16

Notes

Quantitative Phase Analyses and microstructural evolution analysis details 21

Initial structural Rietveld refinement of PXRD 24

References 35

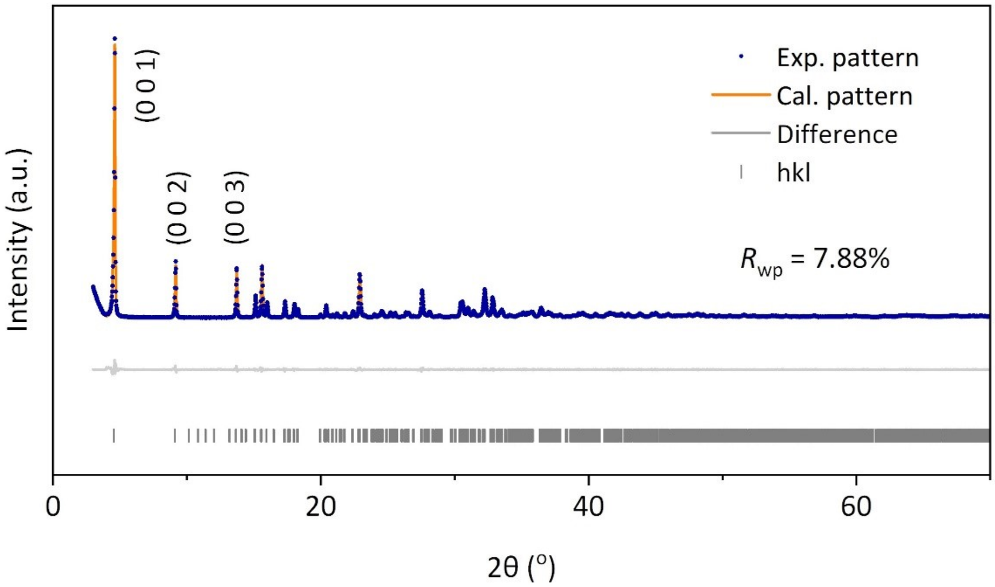

Fig. S1. Pawley refinement of the PXRD for crystalline (S-NEA)$_2$PbBr$_4$, along with patterns simulated from the published Crystallographic Information File (cif) (CCDC 2015618).[1] Experimental data (blue), calculated diffraction pattern (orange), difference (light grey) and symmetry-allowed reflections (grey ticks). X-ray wavelength = 1.5418 Å.

Table S1. Crystallographic data from Pawley refinement of the PXRD for crystalline (S-NEA)$_2$PbBr$_4$.

R_{wp}-value	Space Group	Lattice Parameters	Reported Lattice Parameters
7.88%	$P2_1$	a = 8.7526(5) Å<br>b =7.9557(4) Å<br>c = 19.5021(7) Å<br>α = 90°<br>β = 93.845(5)°<br>γ = 90°	a = 8.75370(19) Å<br>b = 7.95502(16) Å<br>c = 19.5038(5) Å<br>α = 90°<br>β = 93.806(2)°<br>γ = 90°

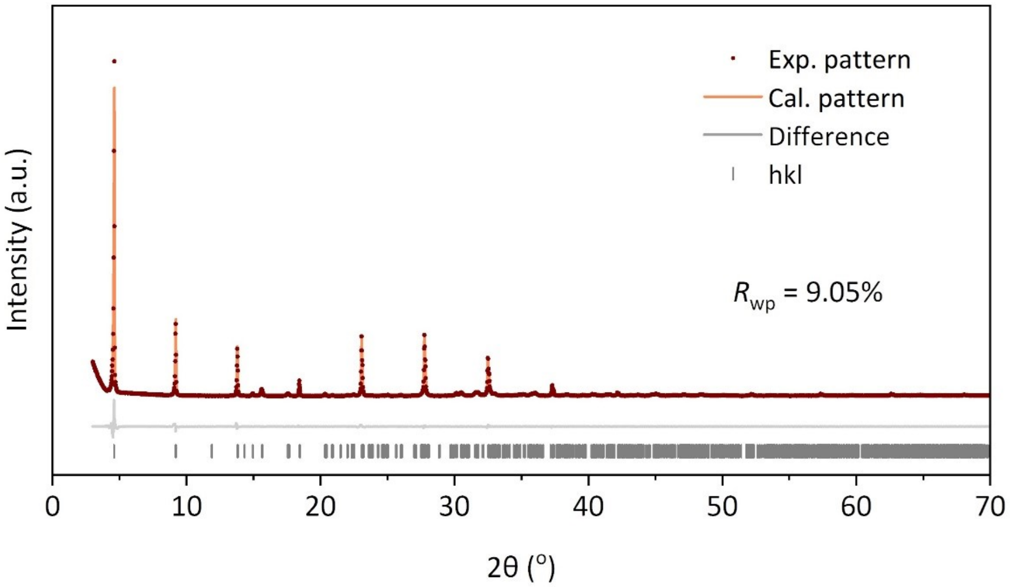

Fig. S2. Pawley refinement of the PXRD for crystalline (*rac*-NEA)$_2$PbBr$_4$, along with patterns simulated from the published cif files (CCDC 2015614)[1]. Experimental data (red), calculated diffraction pattern (orange), difference function (light grey) and symmetry-allowed reflections (grey ticks). X-ray wavelength = 1.5418 Å.

Table S2. Crystallographic data from Pawley refinement of the PXRD for crystalline (*rac*-NEA)$_2$PbBr$_4$.

R_{wp}-value	Space Group	Lattice Parameters	Reported Lattice Parameters
9.05%	$P2_1/c$	a = 19.2789(3) Å<br>b = 8.0848(2) Å<br>c = 8.7393(2) Å<br>α = 90°<br>β = 90.583(35)°<br>γ = 90°	a = 19.2528(9) Å<br>b = 8.0769(4) Å<br>c = 8.7280(5) Å<br>α = 90°<br>β = 90.281(3)°<br>γ = 90°

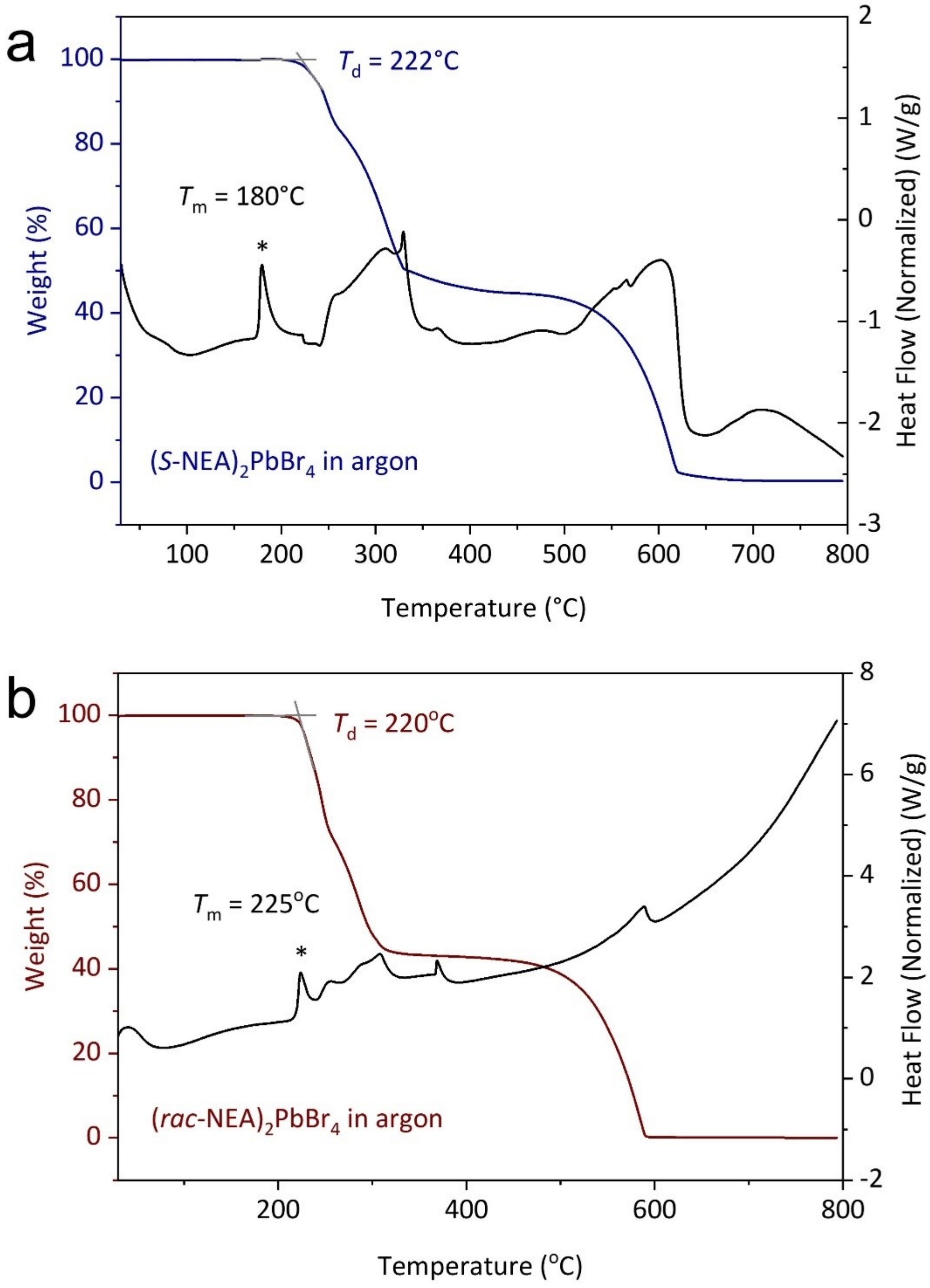

Fig. S3. Simultaneous DSC-TGA experiment in argon: (a) the as-synthesised crystalline (*S*-NEA)$_2$PbBr$_4$, showing a decomposition temperature of *ca*. 222°C and a melting temperature of *ca*. 180°C; (b) the as-synthesised crystalline (*rac*-NEA)$_2$PbBr$_4$, showing a decomposition temperature of *ca*. 220°C and a melting point of *ca*. 225°C. All heating rates were 10 °C min^{-1}.

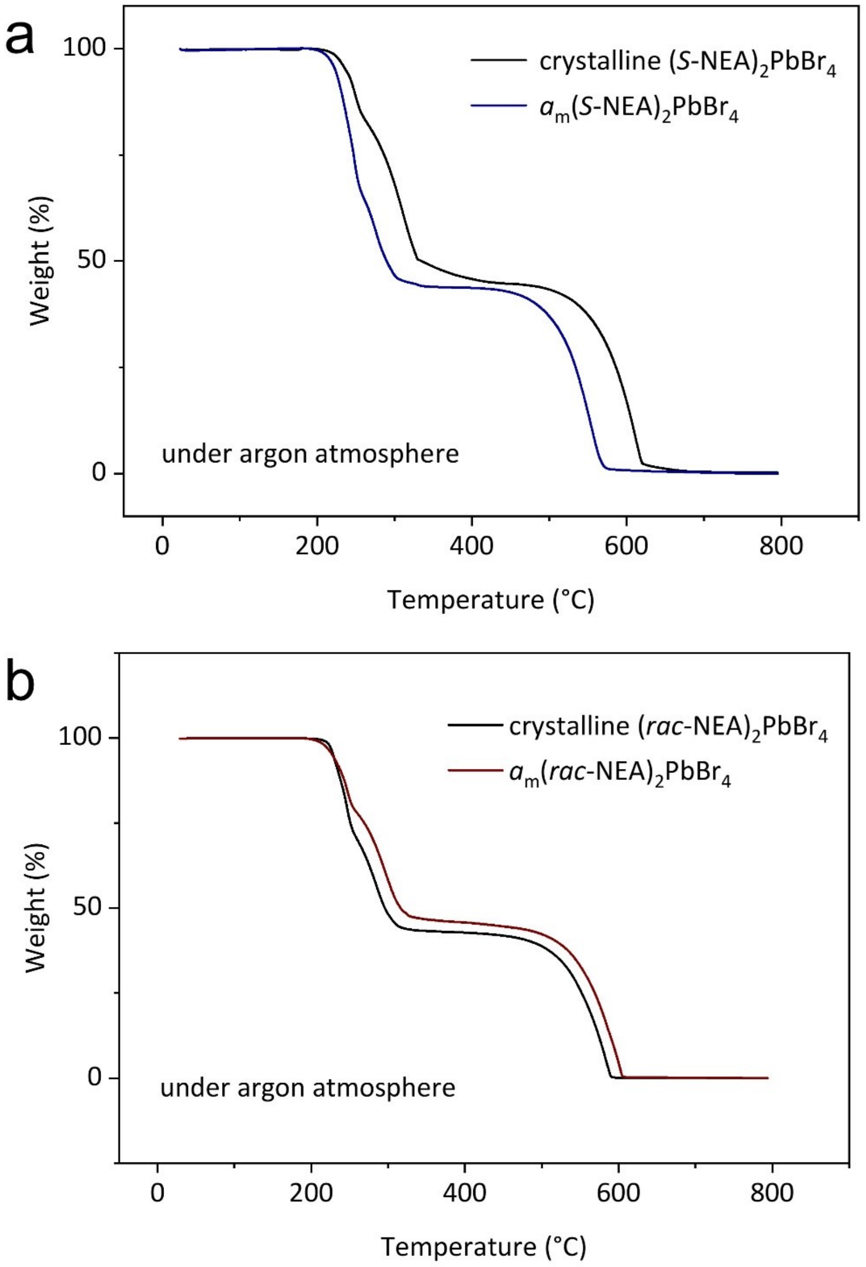

Fig. S4. TGA profiles of (a) (S-NEA)$_2$PbBr$_4$ and (b) (rac-NEA)$_2$PbBr$_4$ in argon before and after ball-milling. All heating rates were 10 ^{o}C min^{-1}.

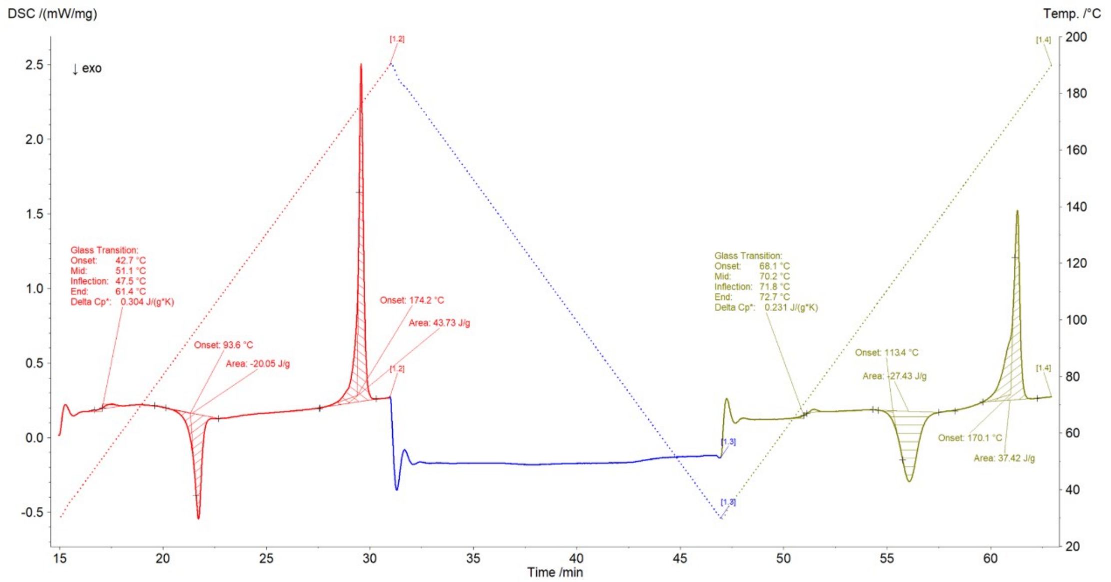

Fig. S5. Full DSC scan of a_m(*S*-NEA)$_2$PbBr$_4$, where the sample was heated to 190°C at 10 ^{o}C min^{-1}, cooled to 30°C at 10 ^{o}C min^{-1}, then heated again to 190°C at 10°C min^{-1}. In the first heating upscan, the glass transition temperature of a_m(*S*-NEA)$_2$PbBr$_4$ was recorded as 51°C and the crystallisation temperature was recorded as 94°C. In the second heating upscan, the glass transition temperature of a_g(*S*-NEA)$_2$PbBr$_4$ (a_g: melt-quenched glass) was recorded as 70°C, coincident with the literature value.[2]

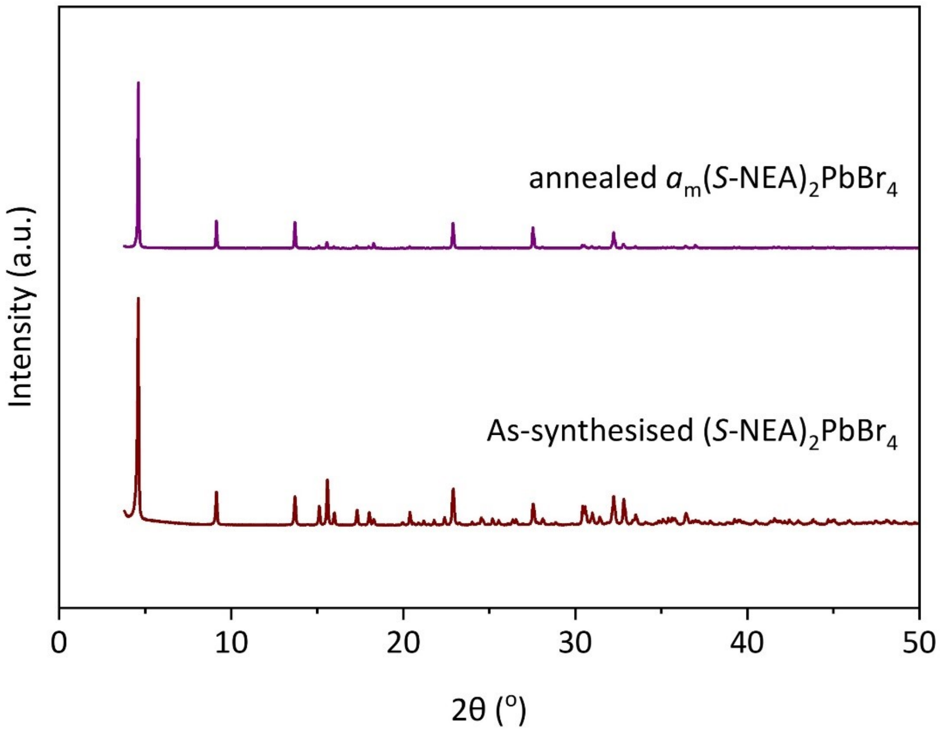

Fig. S6. PXRD pattern of the as-synthesised crystalline (*S*-NEA)$_2$PbBr$_4$ and annealed a_m(*S*-NEA)$_2$PbBr$_4$ after heating at 120°C for 5 minutes, showing complete crystallisation. X-ray wavelength = 1.5418 Å.

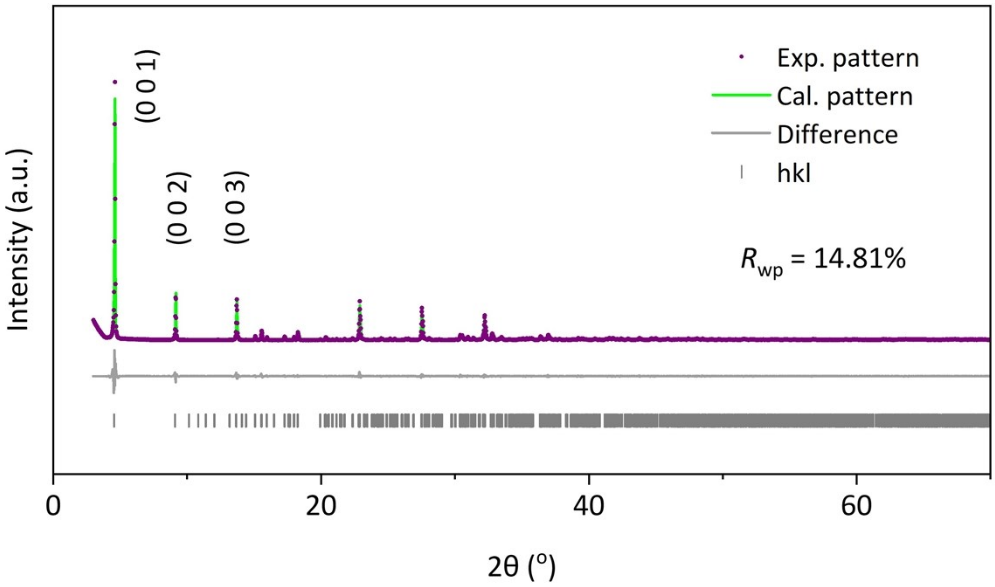

Fig. S7. Pawley refinement of the PXRD for annealed (S-NEA)$_2$PbBr$_4$ sample in Fig. S6, along with patterns simulated from the published Crystallographic Information File (cif) (CCDC 2015618).[1] Experimental data (purple), calculated diffraction pattern (green), difference (light grey) and symmetry-allowed reflections (grey ticks). X-ray wavelength = 1.5418 Å.

Table S3. Crystallographic data from Pawley refinement of the PXRD for annealed (S-NEA)$_2$PbBr$_4$.

R_{wp}-value	Space Group	Lattice Parameters	Reported Lattice Parameters
14.81%	$P2_1$	a = 8.7635(7) Å<br>b =7.9708(6) Å<br>c = 19.5130(6) Å<br>α = 90°<br>β = 93.860(8)°<br>γ = 90°	a = 8.75370(19) Å<br>b = 7.95502(16) Å<br>c = 19.5038(5) Å<br>α = 90°<br>β = 93.806(2)°<br>γ = 90°

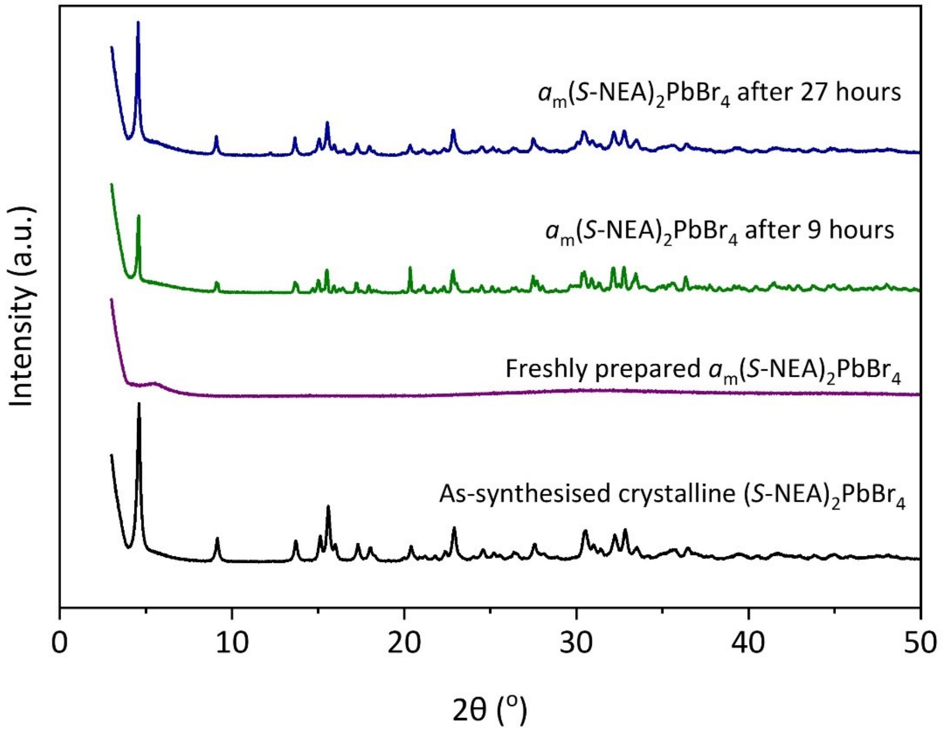

Fig. S8. PXRD pattern of the as-synthesised crystalline (*S*-NEA)$_2$PbBr$_4$, the freshly prepared a_m(*S*-NEA)$_2$PbBr$_4$ and the a_m(*S*-NEA)$_2$PbBr$_4$ products stored in an ambient environment over a period of time, showing that a_m(*S*-NEA)$_2$PbBr$_4$ crystallised within 9 hours when stored in air. X-ray wavelength = 1.5418 Å.

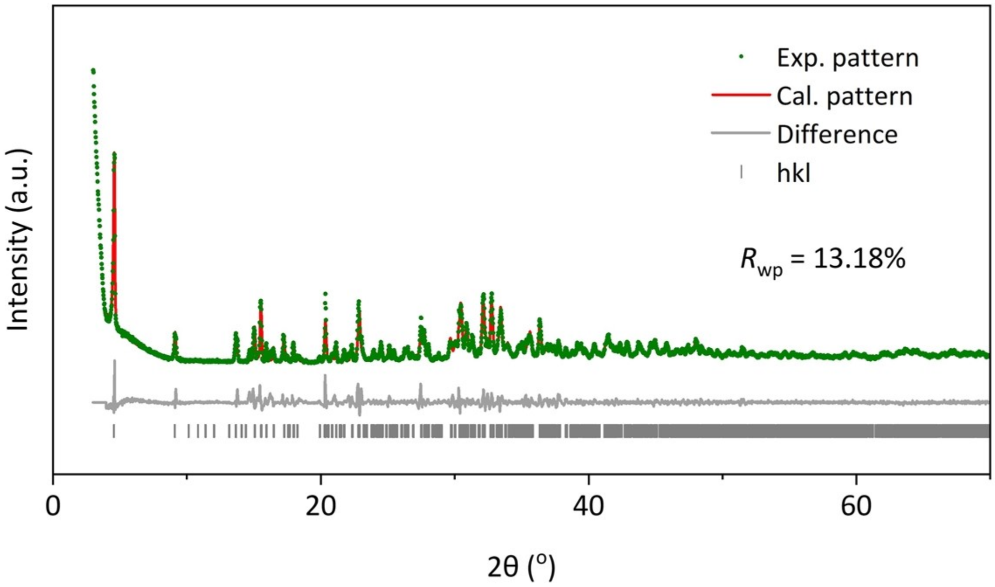

Fig. S9. Pawley refinement of the PXRD for recrystallised a_m(*S*-NEA)$_2$PbBr$_4$ product after 9 hours stored in an ambient environment in Fig. S8, along with patterns simulated from the published Crystallographic Information File (cif) (CCDC 2015618).[1] Experimental data (green), calculated diffraction pattern (red), difference (light grey) and symmetry-allowed reflections (grey ticks). X-ray wavelength = 1.5418 Å.

Table S4. Crystallographic data from Pawley refinement of the PXRD for recrystallised a_m(*S*-NEA)$_2$PbBr$_4$ product after 9 hours stored in an ambient environment.

R_{wp}-value	Space Group	Lattice Parameters	Reported Lattice Parameters
13.18%	$P2_1$	a = 8.7666(12) Å<br>b =7.9856(12) Å<br>c = 19.5324(26) Å<br>α = 90°<br>β = 93.894(12)°<br>γ = 90°	a = 8.75370(19) Å<br>b = 7.95502(16) Å<br>c = 19.5038(5) Å<br>α = 90°<br>β = 93.806(2)°<br>γ = 90°

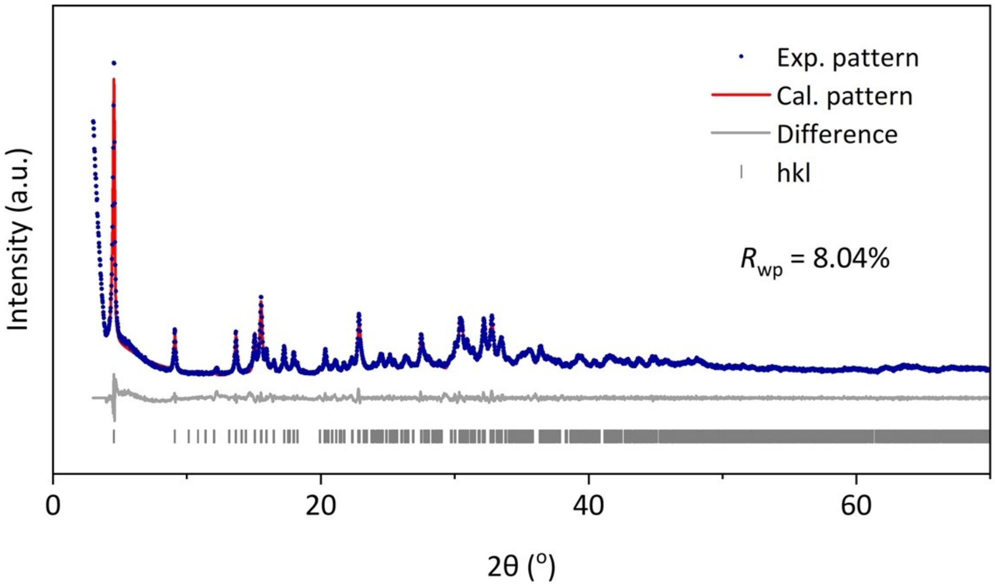

Fig. S10. Pawley refinement of the recrystallised $\alpha_m(S\text{-NEA})_2PbBr_4$ product after 27 hours stored in an ambient environment in Fig. S8, along with patterns simulated from the published Crystallographic Information File (cif) (CCDC 2015618).[1] Experimental data (blue), calculated diffraction pattern (red), difference (light grey) and symmetry-allowed reflections (grey ticks). X-ray wavelength = 1.5418 Å.

Table S5. Crystallographic data from Pawley refinement of the PXRD for recrystallised $\alpha_m(S\text{-NEA})_2PbBr_4$ product after 27 hours stored in an ambient environment.

R_{wp}-value	Space Group	Lattice Parameters	Reported Lattice Parameters
8.04%	$P2_1$	a = 8.7593(31) Å<br>b =7.9633(23) Å<br>c = 19.5025(37) Å<br>α = 90°<br>β = 93.920(21)°<br>γ = 90°	a = 8.75370(19) Å<br>b = 7.95502(16) Å<br>c = 19.5038(5) Å<br>α = 90°<br>β = 93.806(2)°<br>γ = 90°

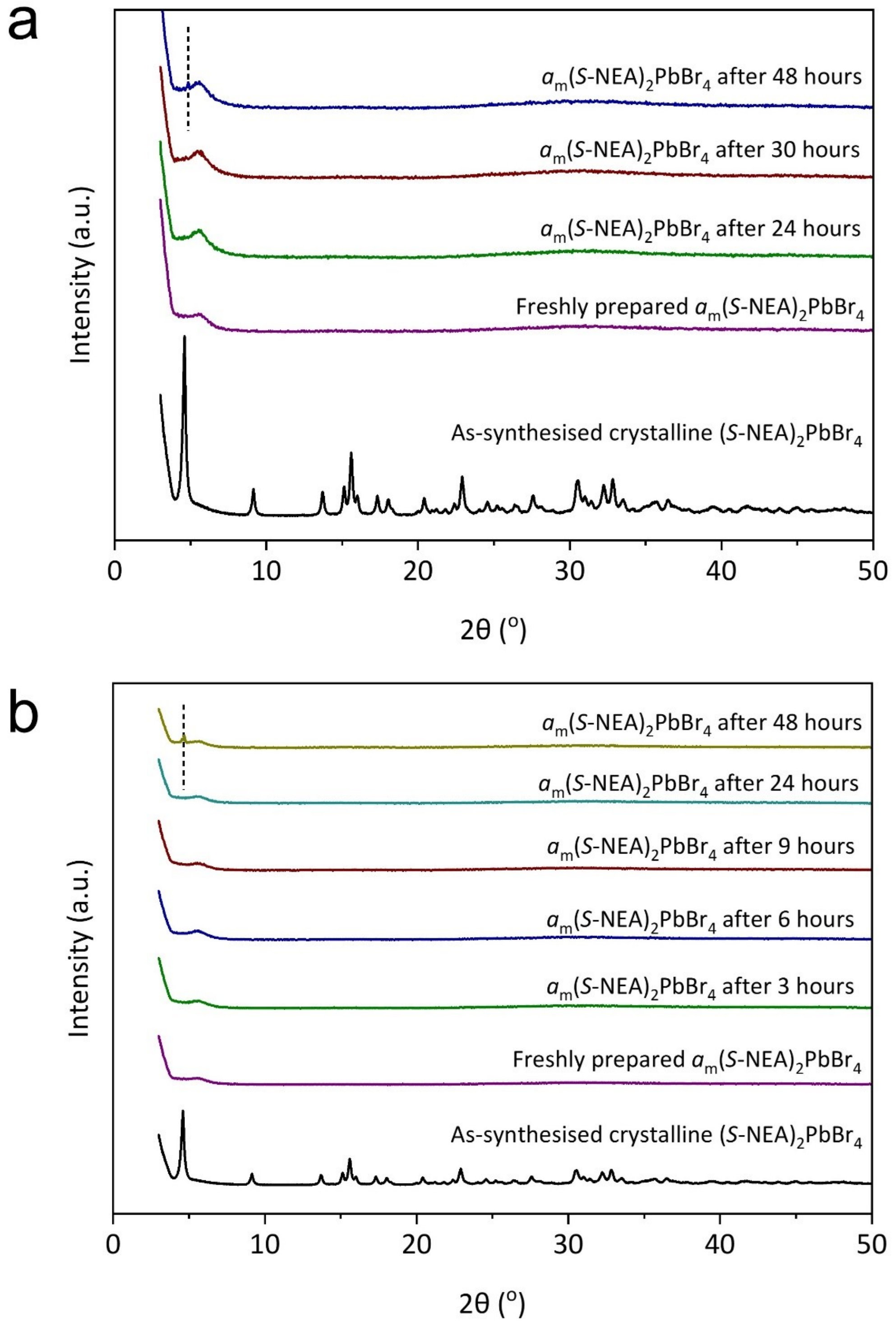

Fig. S11. PXRD pattern of the as-synthesised crystalline (*S*-NEA)$_2$PbBr$_4$, the freshly prepared a_m(*S*-NEA)$_2$PbBr$_4$ and a_m(*S*-NEA)$_2$PbBr$_4$ products when stored (a) in a freezer at *ca*. 0°C or (b) under vacuum over a period of time. The dashed lines show the position of the strongest Bragg peak in the as-synthesised crystalline (*S*-NEA)$_2$PbBr$_4$. X-ray wavelength = 1.5418 Å.

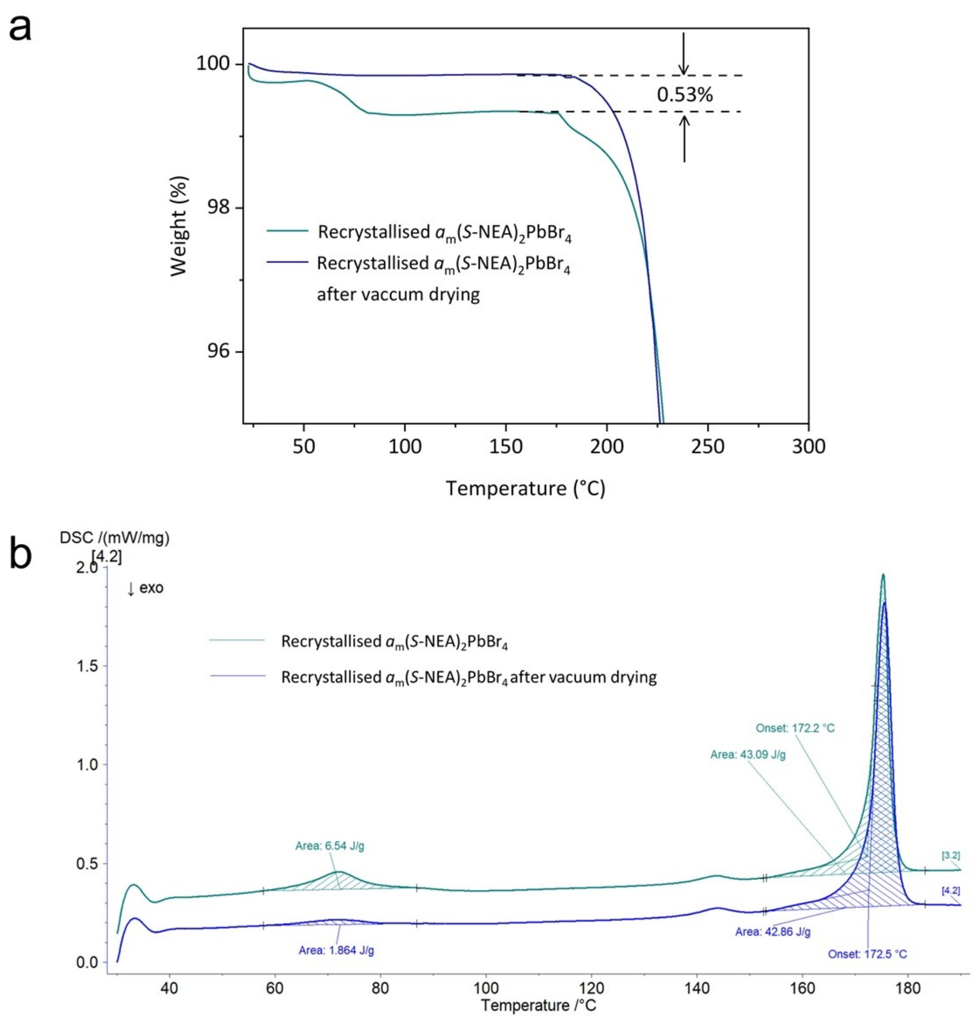

Fig. S12. (a) TGA and (b) DSC profiles of the recrystallised $a_m(S\text{-}NEA)_2PbBr_4$ sample after two days stored in an ambient environment and the same batch of sample that was then vacuum dried at 90°C for 2 hours. The recrystallised sample without further vacuum drying showed a minor mass loss of 0.53% at around 70°C in TGA and its DSC profile had an endothermic peak at around the same temperature region. After being vacuum dried at 90°C for 2 hours, these features almost disappear, indicating that the sample recrystallised in an ambient environment might have absorbed moisture from air.

Table S6. CHN microanalysis of crystalline (*S*-NEA)$_2$PbBr$_4$ and a_m(*S*-NEA)$_2$PbBr$_4$.

**Substance**	**Weight taken (mg)**	**C (%)**	**H (%)**	**N (%)**
Theoretical	-	33.08	3.24	3.22
crystalline (*S*-NEA)$_2$PbBr$_4$ (1)*	2.1766	33.80	3.27	3.29
crystalline (*S*-NEA)$_2$PbBr$_4$ (2)*	1.7610	33.79	3.25	3.26
Mean (ESD)	-	33.80 (0.01)	3.26 (0.01)	3.28 (0.02)
a_m (*S*-NEA)$_2$PbBr$_4$ (1)	2.6184	33.72	3.28	3.34
a_m (*S*-NEA)$_2$PbBr$_4$ (2)	2.5119	33.66	3.29	3.33
Mean (ESD)	-	33.69 (0.04)	3.29 (0.01)	3.34 (0.01)

*(1) and (2) indicate that two measurements were collected per sample.

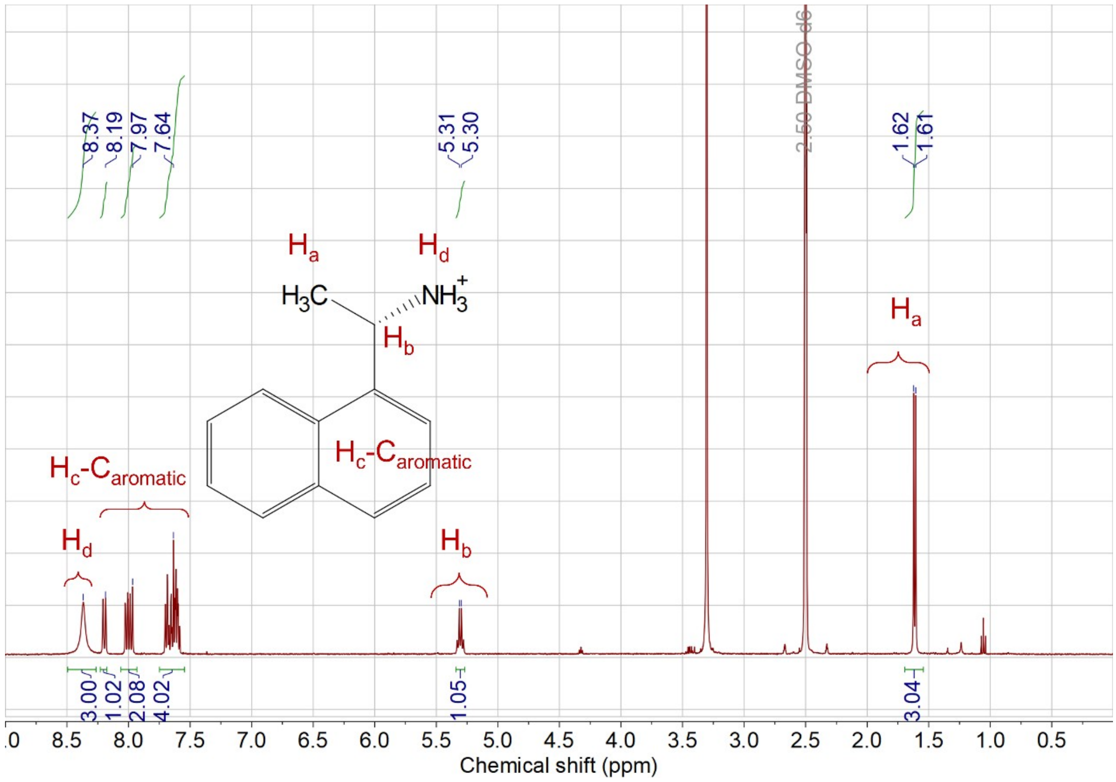

Fig. S13. ^{1}H nuclear magnetic resonance spectrum of a_m(*S*-NEA)$_2$PbBr$_4$.

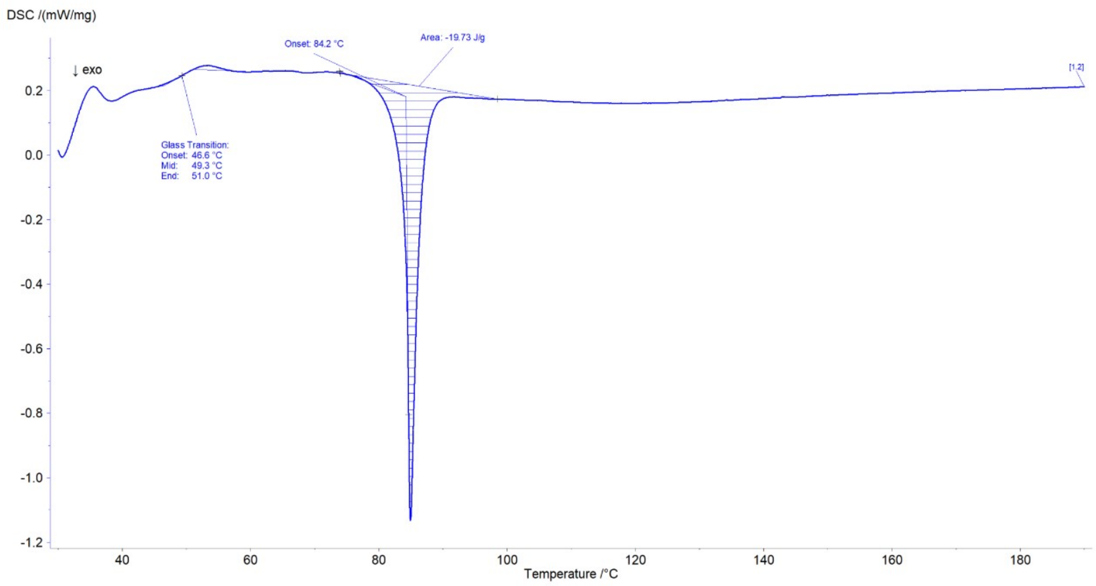

Fig. S14. Full DSC scan of a_m(*rac*-NEA)$_2$PbBr$_4$, where the sample was heated to 190°C at 10 ^{o}C min^{-1} in argon. The glass transition temperature of a_m(*rac*-NEA)$_2$PbBr$_4$ was recorded as 49°C and the crystallisation temperature was recorded as 84°C.

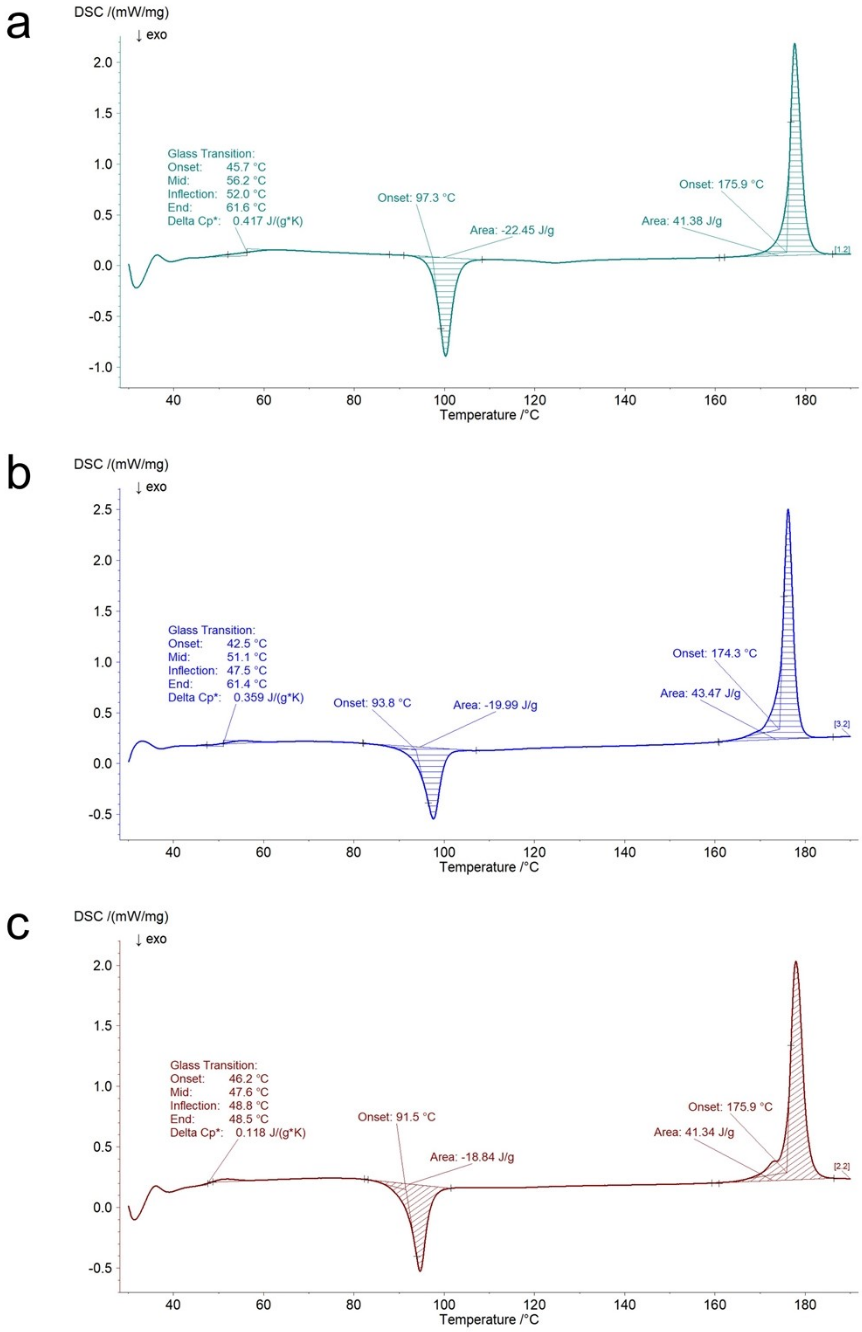

Fig. S15. Full DSC scan of (a) a_m(*S*-NEA)$_2$PbBr$_4$-10 (green), (b) a_m(*S*-NEA)$_2$PbBr$_4$-30 (blue) and (c) a_m(*S*-NEA)$_2$PbBr$_4$-60 (brown), where the sample was heated to 190°C at 10 ^{o}C min^{-1}. Their glass transition temperatures were recorded as 56°C, 51°C and 48°C, respectively. The crystallisation temperature and the enthalpy of crystallisation are 97°C and -22 ± 1 J g^{-1} (a_m(*S*-NEA)$_2$PbBr$_4$-10), 94°C and -20 ± 1 J g^{-1} (a_m(*S*-NEA)$_2$PbBr$_4$-30), 92°C and -19 ± 1 J g^{-1} (a_m(*S*-NEA)$_2$PbBr$_4$-60), respectively. The melting temperature and the enthalpy of the recrystallised products are recorded as 176°C and 41 ± 1 J g^{-1} (a_m(*S*-NEA)$_2$PbBr$_4$-10), 174°C and 43 ± 1 J g^{-1} (a_m(*S*-NEA)$_2$PbBr$_4$-30), 176°C and 41 ± 1 J g^{-1} (a_m(*S*-NEA)$_2$PbBr$_4$-60), respectively.

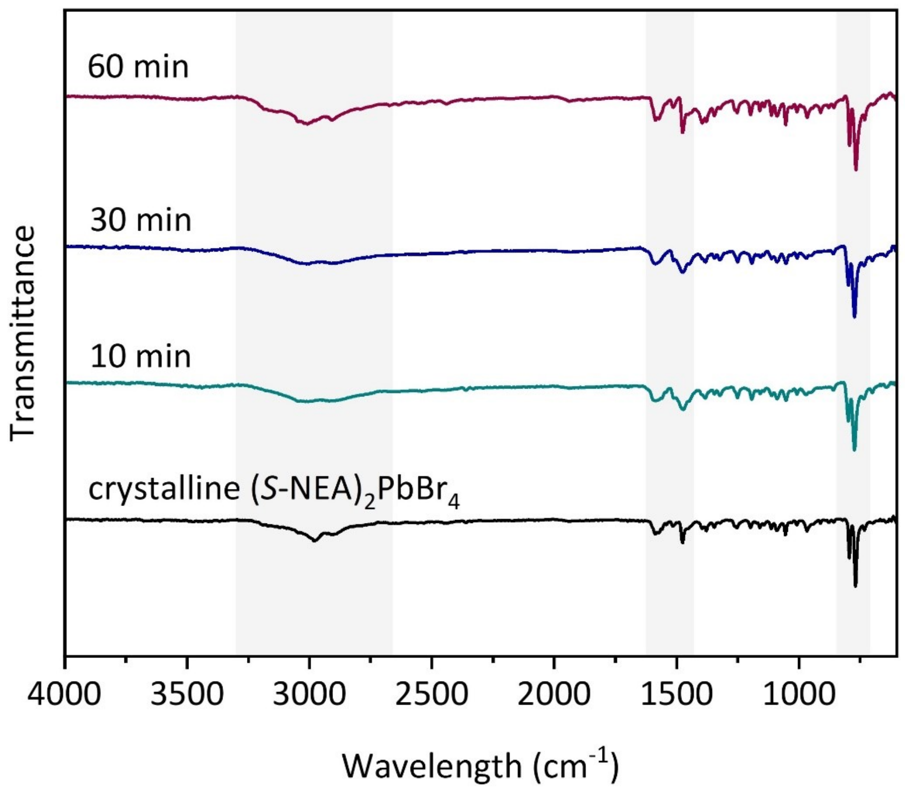

Fig. S16. FT-IR of the as-synthesised crystalline (S-NEA)$_2$PbBr$_4$ and a_m(S-NEA)$_2$PbBr$_4$ with different milling times. The regions exhibiting peaks of interest are shaded grey.

Quantitative Phase Analyses (QPA) and microstructural evolution analysis details

Rietveld QPA of experimental diffraction patterns was performed using the TOPAS-Academic V7 software.[3] The structural model of (S-NEA)$_2$PbBr$_4$ was refined on PXRD data obtained from a laboratory X-ray diffraction instrument (see following section). The scattering signal out of a_m(*S*-NEA)$_2$PbBr$_4$ was modelled with a single Gaussian peak. The structural models of tetragonal and orthorhombic lead oxide minimum, which were found as minor impurities in the powder mix, were retrieved from the Inorganic Crystal Structure Database.[4] The in-situ PXRD dataset under milling conditions was fit sequentially, with a convergence criterion of 0.001 and a maximum number of iterations of 10000. An experimental, fixed background profile measured on an empty section of the jar before milling, was subtracted by fitting a background scale parameter together with a 2 terms polynomial function. As described elsewhere,[5,6] the sample powders inside the milling jar are distributed across different locations, e.g. some powders float randomly inside the jar, while some are more attached to the walls of the jar, which accordingly leads to a range of distances from the sample to the detector. As such, diffraction with the ball-milling setup results in splitting of each Bragg reflection into a convolution of 2θ positions. The difference in 2θ angle can be minimised when the jar is accurately aligned, with negligible scattering contribution from the sample distributed within the jar. Therefore, in our work, the crystal structure model of each compound was introduced twice ("top phase" and "bottom phase" from here on) with two modified Thompson-Cox-Hastings pseudo-Voigt functions (TCHZ) peak shape functions (with fixed parameters as refined on the Si standard) and independent scale parameters.

As described by Lampronti et al.,[5,6] the dependence of the profile full-width-at-half-max (FWHM) on 2θ was described with a modified TCHZ function where U, V, W (Gaussian) and X (Lorenzian) are the parameters that have been refined in the current case.

$$TCHZ = \eta\Gamma_L + (1-\eta)\Gamma_G \quad \text{Equation S1}$$

$$\Gamma_G = \sqrt{\left(Utan^2\theta + Vtan\theta + W + Z/cos^2\theta\right)} \quad \text{Equation S2}$$

$$\Gamma_L = \frac{X}{cos\theta} + Ytan\theta \quad \text{Equation S3}$$

$$\eta = 1.336603\ q - 0.47719\ q^2 + 0.1116\ q^3 \qquad \text{Equation S4}$$

$$q = \Gamma_L / \Gamma \qquad \text{Equation S5}$$

$$\Gamma = \sqrt{\Gamma_G^5 + A\Gamma_G^4\Gamma_L + B\Gamma_G^3\Gamma_L^2 + C\Gamma_G^2\Gamma_L^3 + D\Gamma_G\Gamma_L^4 + \Gamma_L^5} = FWHM \qquad \text{Equation S6}$$

$$A = 2.69269;\ B = 2.42843; C = 4.47163; D = 0.07842 \qquad \text{Equation S7}$$

Where η is the Pseudo-Voigt mixing parameter, and Γ_G and $\Gamma_L$are the Gaussian and Lorentzian full width half maxima, respectively. Additionally, to model the evident peak asymmetry, the TCHZ was further split to differentiate the contribution at the left-hand side and at the right-hand site to the overall peak profile for the inner and outer scattering vectors $\vec{s}_1$ and $\vec{s}_3$. The final $TCHZ_{split}$ function is applied to describe the overall dependence of the FWHM according to Equation S7,

$$TCHZ_{split} = \left(\eta\Gamma_L + (1-\eta)\Gamma_G\right)_{Left} + \left(\eta\Gamma_L + (1-\eta)\Gamma_G\right)_{Right} \qquad \text{Equation S7}$$

Microstructural investigations were performed assuming that the sample contribution to peak broadening was related to size only. A Lorentzian function was convoluted for each phase, with a single isotropic Crystal Size (CS) parameter related to Γ_L as in the Scherrer equation (equations S8 and S9),[7]

$$L(nm) = \frac{K_s\lambda}{(cos\theta) * 10 * \tau} \qquad \text{Equation S8}$$

$$\Gamma_L = \frac{57.32 * \lambda}{cos\theta * CS} \qquad \text{Equation S9}$$

in which, L is the mean size of the ordered (crystalline) domains, K_s is a shape factor constant in the range (typically 0.9), λ is the X-ray wavelength, τ is the peak width in radians at FWHM. The top and bottom phases of the same compound were constrained to have the same CS parameter. We here remind that the estimated standard deviation (ESD) from the Rietveld calculation has no bearing on the precision

or accuracy, but is merely related to the mathematical fit of the model.[8] In other words, absolute numbers have a degree of uncertainty that cannot really be measured. On the other hand, so long as the same approach is used for all scans within a dataset, trends are reliable. For what concerns the accuracy of the size determination, it is known that for a typical laboratory X-ray diffraction instrument the Scherrer analysis provides sensitivity to crystallite size in the 1-100 nm range, the upper limit being set by the instrumental broadening.[9] This also means that the smaller the crystal size, the less the Scherrer size value is affected by how the instrumental broadening is defined. For example, modeling the instrumental contribution for the analyzed datasets with a single conventional TCHZ function, instead of two split TCHZ functions, has a more significant effect on the estimated CS for larger CSs. To summarize, the smaller the crystal size, the more reliable the number. It is also important to note that the peak shape tends to be dominated by the larger crystallites rather than the smaller ones, so the calculated size tends to be overestimated.[9] The scale factors of the crystalline and amorphous phases were normalized using their respective maximum scale factor, i.e. the scale factor from the refinement of the first pattern for the crystalline phase, and the scale factor from the refinement of the last pattern for the amorphous phase. For each refinement the weight percentage of each of the two phases was calculated as the ratio between its normalized scale factor and the sum of the two normalized scale factors times 100. Error propagation rules were applied accordingly to calculate the estimated standard deviation.

While the visual inspection of a Rietveld plot is the most reliable way to determine the quality of a fit, this is not practical for large datasets, such as those presented here. A global check of a sequential refinement can be efficiently performed by comparing a number of "goodness of fit" indices. One is the weighted profile R-factor (R_{wp}),

$$R_{wp}^{2} = \frac{\sum_{i} w_i (y_{c,i} - y_{o,i})^2}{\sum_{i} w_i (y_{o,i})^2}$$

Equation S10

Where y_c and y_o represent the calculated and observed intensity respectively for each point i. and the weight w_i is equal to $1/\sigma^2[y_{o,i}]$. The second index is "chi squared":

$$\chi^2 = \left(\frac{R_{wp}}{R_{exp}}\right)^2$$ Equation S11

where (R_{exp}), the “expected *R* factor”, is:

$$R_{exp}^{\ 2} = \frac{N}{\sum_i w_i (y_{o,i})^2}$$ Equation S12

with *N* as the number of data points.

Initial structural Rietveld refinement of PXRD

The structural model of (S-NEA)$_2$PbBr$_4$ was refined on PXRD data obtained from a laboratory X-ray diffraction instrument using TOPAS-Academic V7.[3] A Chebyshev polynomial function with ten parameter was used to fit the background. The position and orientation of the organic molecules were optimized as rigid bodies. The position of the inorganic ions was refined with no constraints. One thermal parameter was applied for each atomic species. The Pseudo-Voigt function used to model the peak shape and the parameters describing the diffractometer geometry were first optimized using an LaB$_6$ standard with a fundamental parameter approach.[10] These were fixed for the structural refinements, while one further isotropic parameter was used to take into account the sample Lorentzian contribution to peak broadening (see previous section). The March-Dollase model for preferred orientation was applied on the (0 0 1) crystallographic direction. The refinement converged with χ^2 and R_{wp} values of 16.72% and 3.56 respectively. The Rietveld refinement plot is shown in Figure S21.

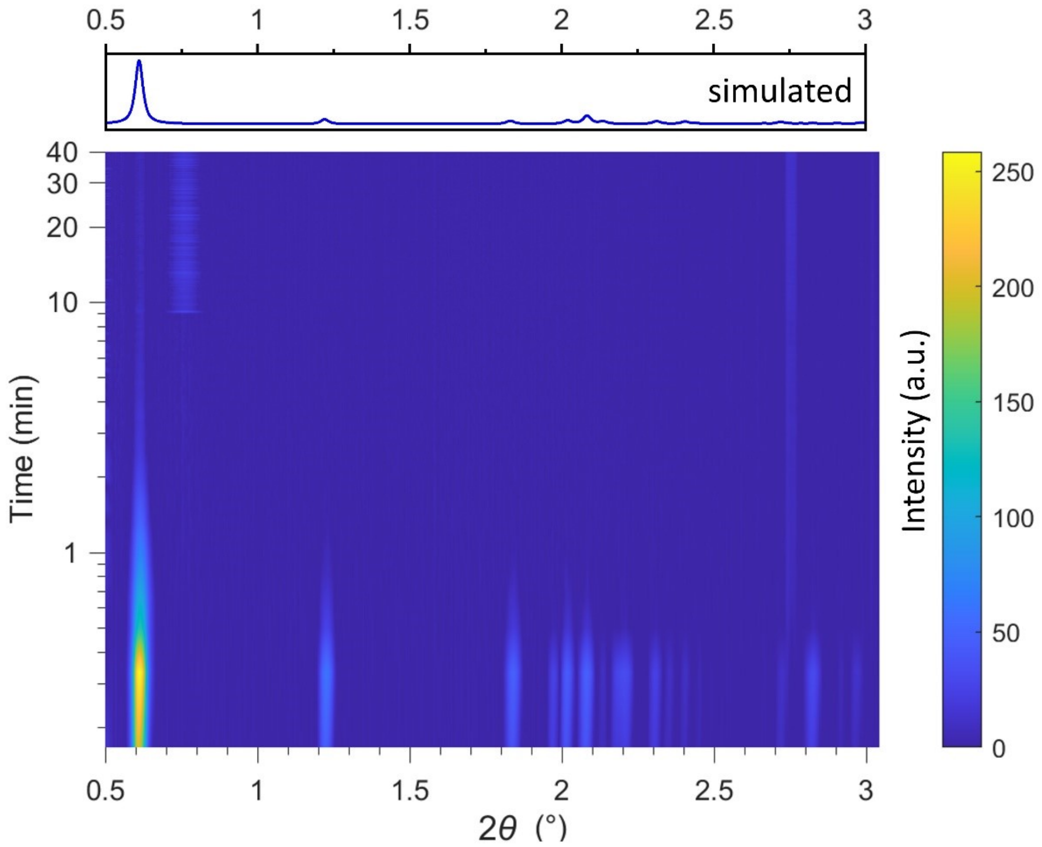

Fig. S17. Full time-resolved in situ X-ray diffractogram for the mechanically-induced amorphisation of (*S*-NEA)$_2$PbBr$_4$. The simulated PXRD pattern of crystalline (*S*-NEA)$_2$PbBr$_4$ is shown above the time-solved diffractogram. The broad band at around 2θ = 0.75° can be attributed to the noise from the PMMA jar. Synchrotron radiation wavelength λ = 0.207351 Å.

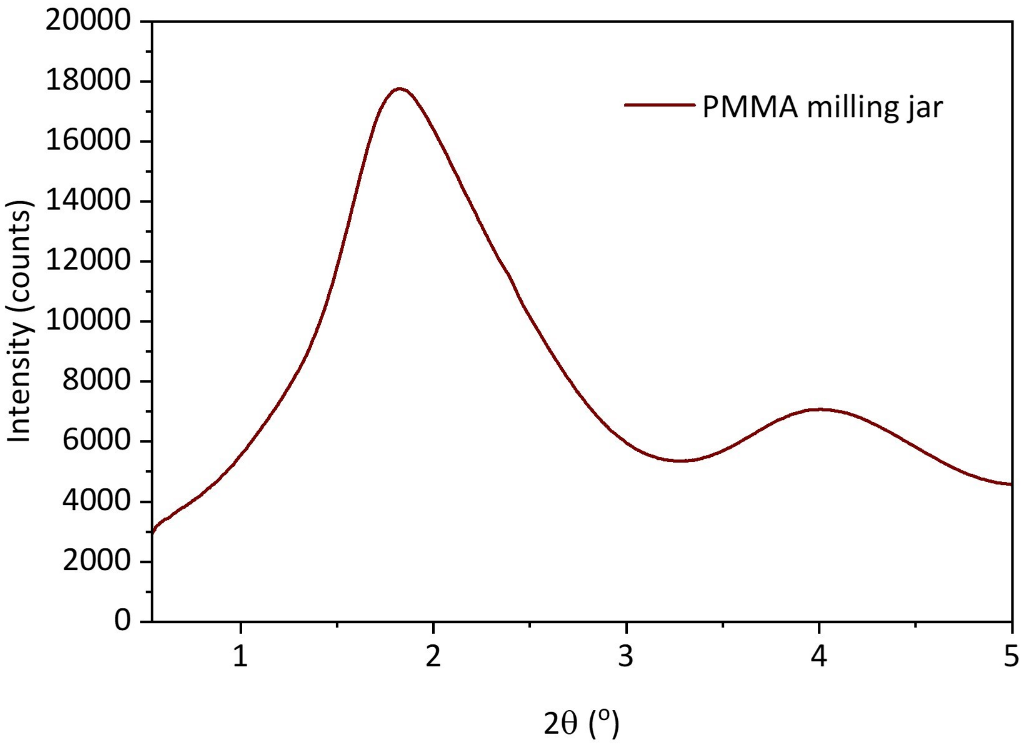

Fig. S18. PXRD pattern of the PMMA milling jar using the diffractogram collected at PETRA-III, showing two broad peaks at approximately 1.8° and 4.1°, respectively. Synchrotron radiation wavelength λ = 0.207351 Å. Synchrotron radiation wavelength λ = 0.207351 Å.

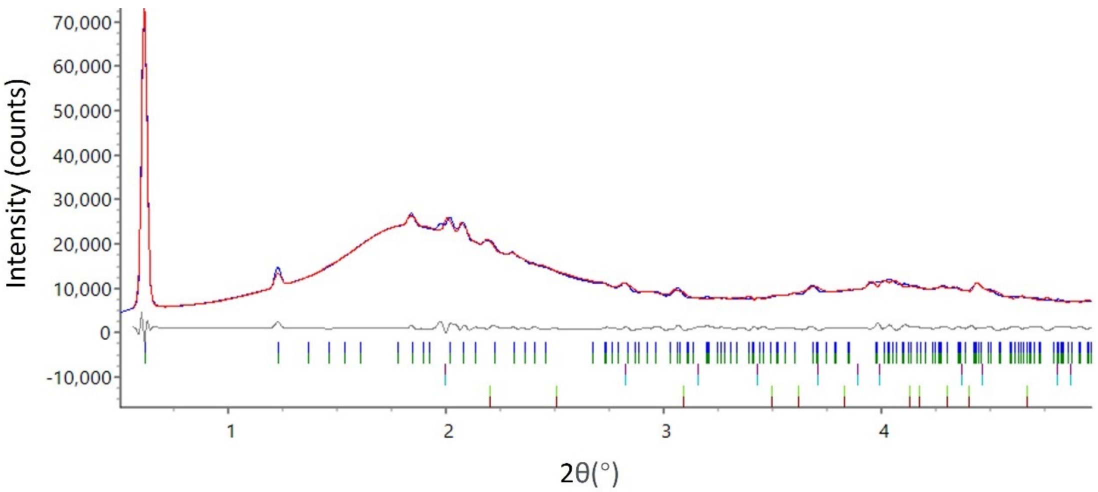

Fig. S19. An example Rietveld fit using the X-ray diffractograms collected at PETRA-III, showing experimental (blue line), calculated (red line), and difference (grey line) patterns of (S-NEA)$_2$PbBr$_4$ under milling conditions at time 0. Peak marks are indicated for (S-NEA)$_2$PbBr$_4$ (top), tetragonal minimum, Pb$_3$O$_4$, (middle) and orthorhombic minimum (bottom). Each symmetry-allowed reflections are marked twice, as diffraction with this setup results in splitting of each Bragg reflection into a convolution of 2θ positions. Synchrotron radiation wavelength λ = 0.207351 Å. As shown in Fig. S18, the broad peaks at approximately 1.8° and 4.1° originate from the PMMA milling jar.

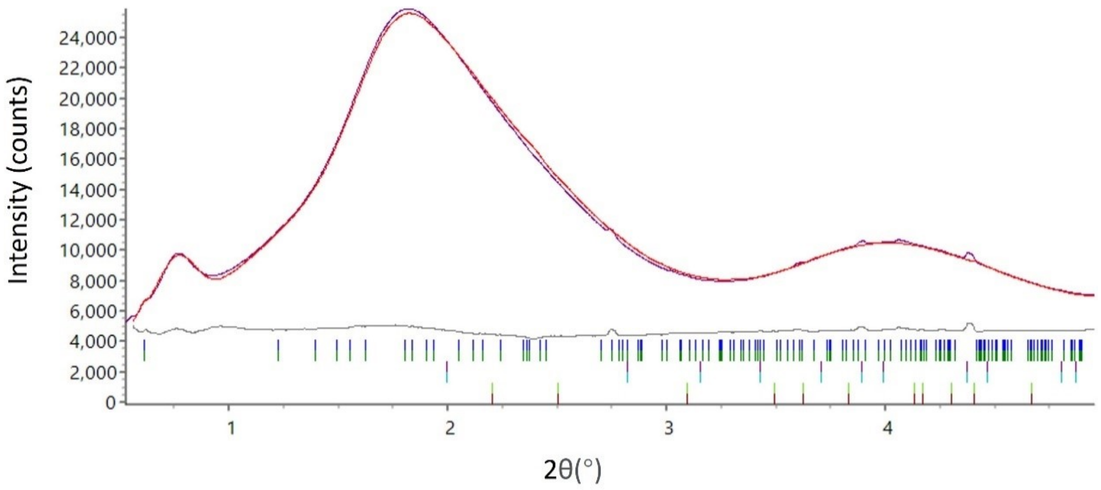

Fig. S20. An example Rietveld fit using the X-ray diffractograms collected at PETRA-III, showing experimental (purple line), calculated (red line), and difference (grey line) patterns of (S-NEA)$_2$PbBr$_4$ under milling conditions after 8 minutes. Peak marks are indicated for (S-NEA)$_2$PbBr$_4$ (top), tetragonal minimum, Pb_3O_4, (middle) and orthorhombic minimum (bottom). Each symmetry-allowed reflections are marked twice, as diffraction with this setup results in splitting of each Bragg reflection into a convolution of 2θ positions. Synchrotron radiation wavelength λ = 0.207351 Å. As shown in Fig. S18, the broad peaks at approximately 1.8° and 4.1° originate from the PMMA milling jar. The small broad peak at approximately 0.8° is attributed to diffuse scattering of the amorphous product.

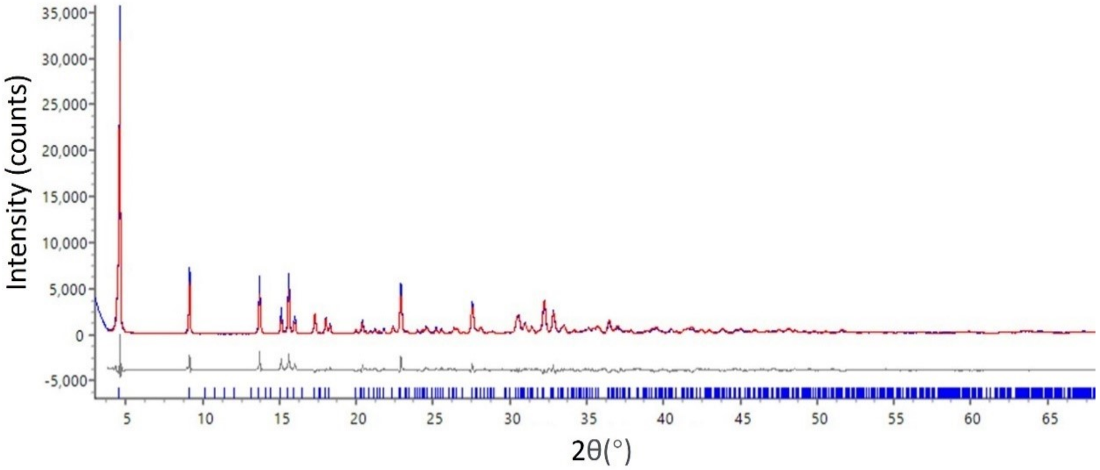

Fig. S21. The initial structural Rietveld refinement of $(S\text{-}NEA)_2PbBr_4$ using the PXRD data obtained from a laboratory X-ray diffraction instrument. Experimental data (blue), calculated diffraction pattern (red), difference (light grey) and symmetry-allowed reflections (blue ticks). X-ray wavelength = 1.5418 Å.

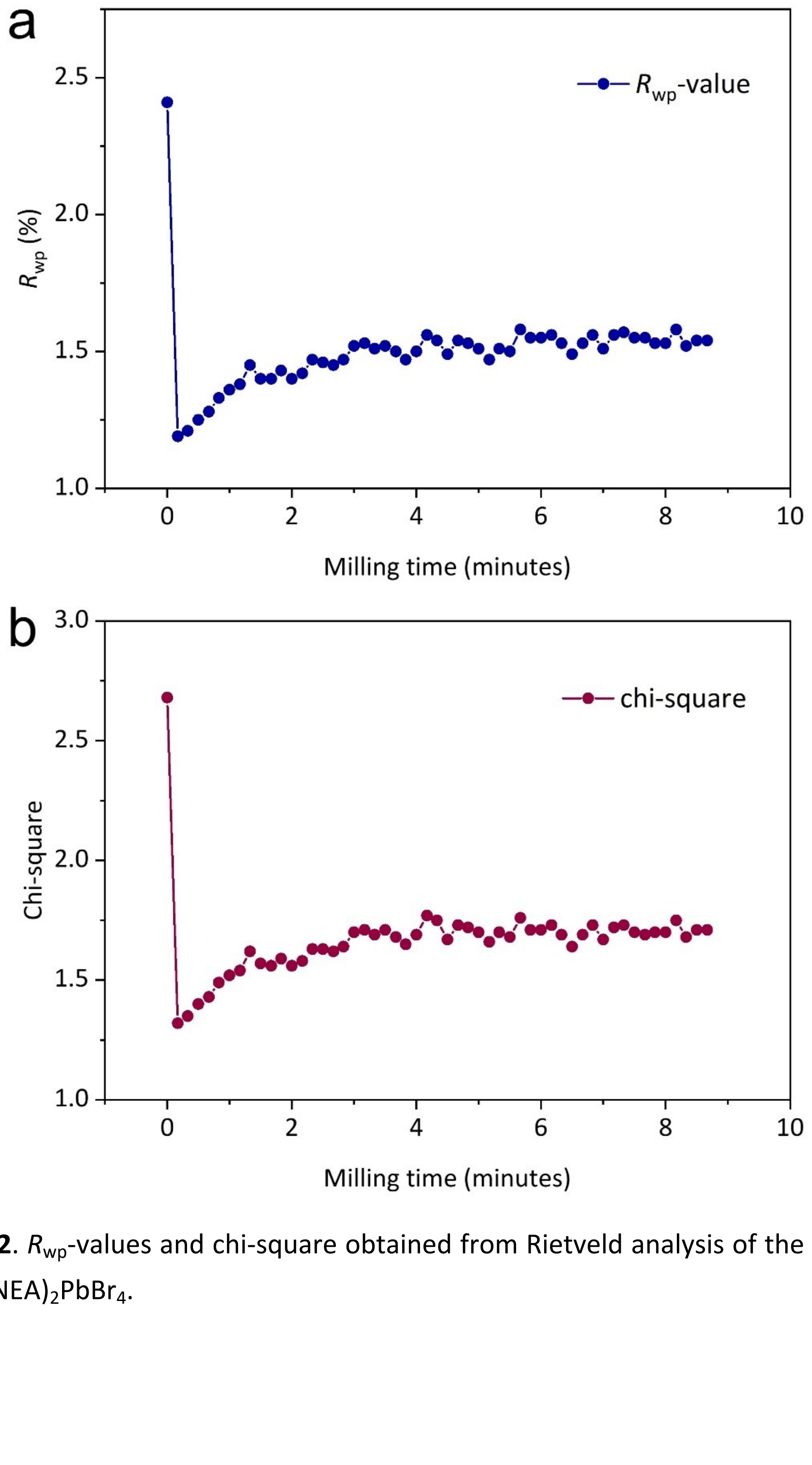

Fig. S22. R_{wp}-values and chi-square obtained from Rietveld analysis of the TRIS PXRD for (S-NEA)$_2$PbBr$_4$.

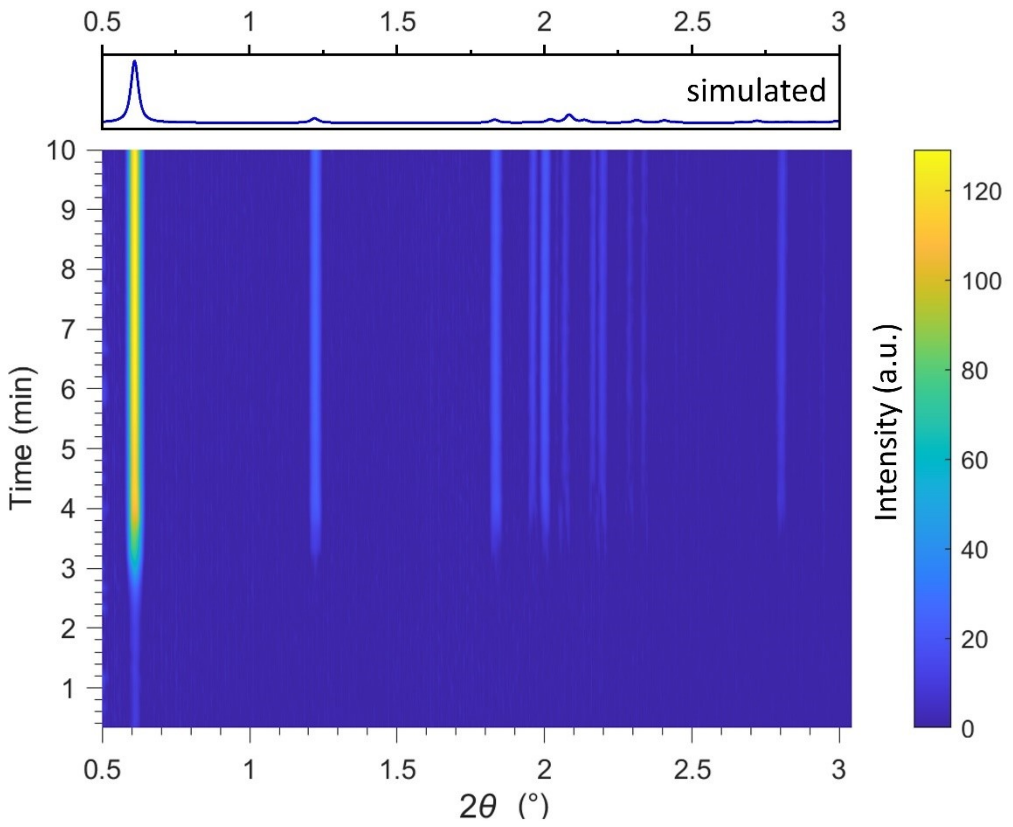

Fig. S23. Full time-resolved in situ X-ray diffractogram of a_m(S-NEA)$_2$PbBr$_4$ upon heating towards 80 °C. The simulated PXRD pattern of crystalline (*S*-NEA)$_2$PbBr$_4$ is shown above the time-solved diffractogram. Synchrotron radiation wavelength λ = 0.207351 Å. It showed complete crystallisation within 5 minutes, when the temperature reached *ca*. 50 °C.

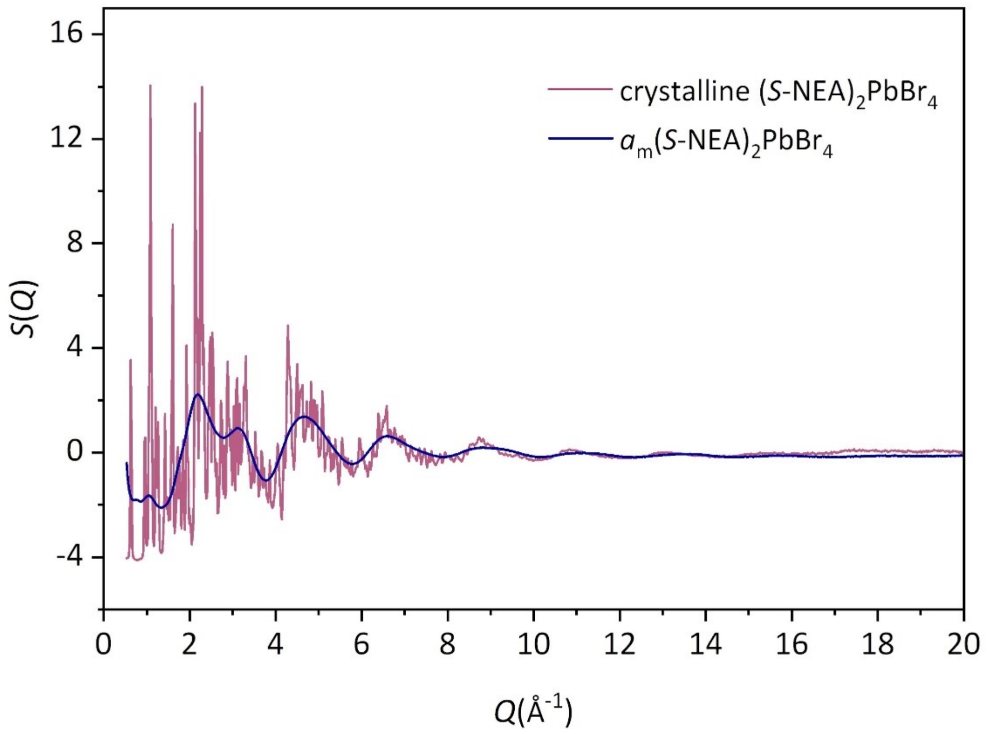

Fig. S24. Structure factor *S*(Q) of crystalline (S-NEA)$_2$PbBr$_4$ (red) and a_m(S-NEA)$_2$PbBr$_4$ (blue).

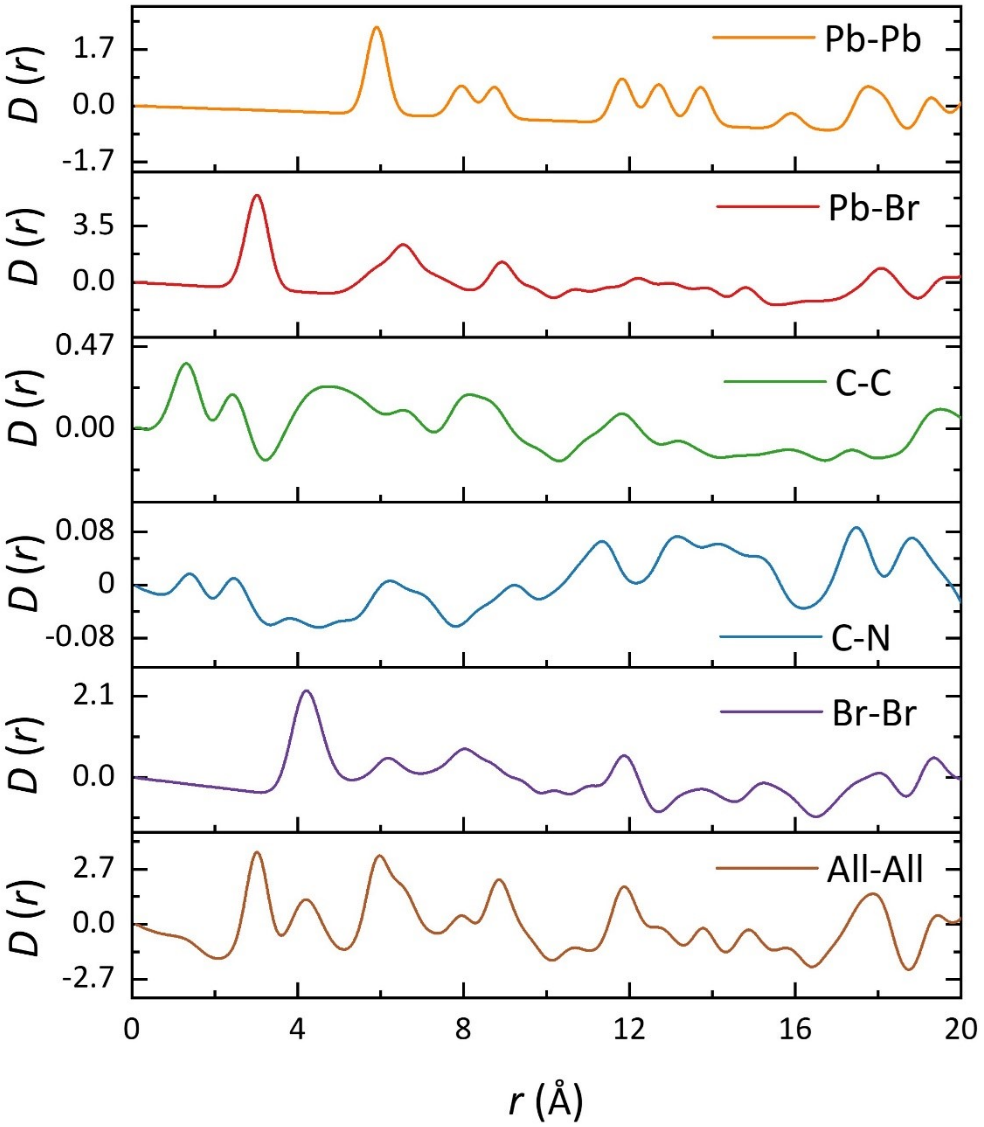

Fig. S25. Selected partial PDFs for (S-NEA)$_2$PbBr$_4$ calculated by PDFgui.[11]

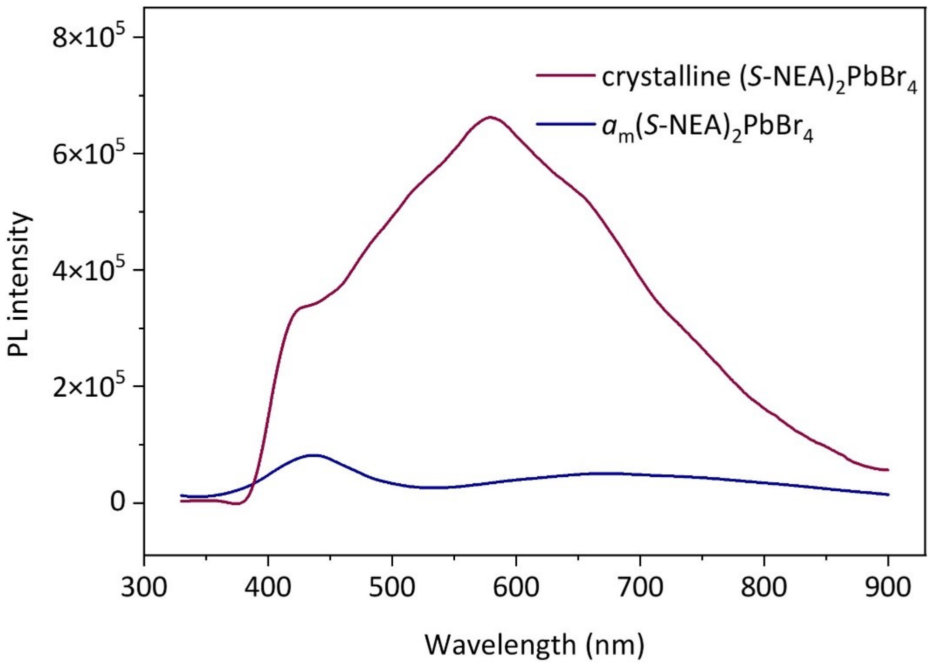

Fig. S26. Steady-state photoluminescence spectra of crystalline (*S*-NEA)$_2$PbBr$_4$ (red) and a_m(*S*-NEA)$_2$PbBr$_4$ (blue) obtained at ambient temperature using a 300 nm light source.

References

1. M. K. Jana, R. Song, H. Liu, D. R. Khanal, S. M. Janke, R. Zhao, C. Liu, Z. Valy Vardeny, V. Blum and D. B. Mitzi, *Nat. Commun.*, 2020, **11**, 4699.
2. A. Singh, M. K. Jana and D. B. Mitzi, *Adv. Mater.*, 2021, **33**, e2005868.
3. A. A. Coelho, *J. Appl. Crystallogr.*, 2018, **51**, 210-218.
4. R. Allmann and R. Hinek, *Acta Crystallogr. Sect. A Found. Crystallogr.*, 2007, **63**, 412-417.
5. G. I. Lampronti, A. A. L. Michalchuk, P. P. Mazzeo, A. M. Belenguer, J. K. M. Sanders, A. Bacchi and F. Emmerling, *Nat. Commun.*, 2021, **12**, 6134.
6. P. P. Mazzeo, G. I. Lampronti, A. A. L. Michalchuk, A. M. Belenguer, A. Bacchi and F. Emmerling, *Faraday Discuss.*, 2023, **241**, 289-305.
7. A. A. Coelho, *TOPAS-Academic Version 6 - Technical Reference*, 2016.
8. I. C. Madsen and N. V. Y. Scarlett, in *Powder Diffraction: Theory and Practice*, ed. R. E. Dinnebier and S. J. L. Billinge, The Royal Society of Chemistry, 2008, **11**, pp. 298-331.
9. E. A. Payzant, in *Principles and Applications of Powder Diffraction*, ed. A. Clearfield, J. H. Reibenspies and B. Nattamai, John Wiley & Sons, Ltd, 2008, **9**, pp. 365-380.
10. R. W. Cheary, A. A. Coelho and J. P. Cline, *J. Res. Natl. Inst. Stand. Technol.*, 2004, **109**, 1-25.
11. C. L. Farrow, P. Juhas, J. W. Liu, D. Bryndin, E. S. Božin, J. Bloch, T. Proffen and S. J. L. Billinge, *J. Phys.: Condens. Matter*, 2007, **19**, 335219.
